# Supplementary material for: Plectin affects cell viscoelasticity at small and large deformations
Source: Biophys J. 2025 Sep 5;125(1):35–53. doi: 10.1016/j.bpj.2025.09.001 (PMC12821017; doi:10.1016/j.bpj.2025.09.001)
Supplement: Document S2. Article plus supporting material [file mmc2.pdf]

Biophysical Journal
Article

Biophysical Society

Plectin affects cell viscoelasticity at small and large deformations

James P. Conboy,[1] Mathilde G. Lettinga,[1] Nicole van Vliet,[1] Lilli Winter,[2] Gerhard Wiche,[3] Fred C. MacKintosh,[4,5,6] and Gijsje H. Koenderink[1,*]

[1]Department of Bionanoscience and Kavli Institute of Nanoscience Delft, Delft University of Technology, Delft, the Netherlands; [2]Department of Cell and Developmental Biology, Center for Anatomy and Cell Biology, Medical University of Vienna, Vienna, Austria; [3]Max Perutz Laboratories, Department of Biochemistry and Cell Biology, University of Vienna, Vienna, Austria; [4]Department of Chemical and Biomolecular Engineering, Rice University, Houston, Texas; [5]Center for Theoretical Biological Physics, Rice University, Houston, Texas; and [6]Department of Chemistry and Department of Physics & Astronomy, Rice University, Houston, Texas

ABSTRACT Plectin is a giant protein of the plakin family that cross-links the cytoskeleton of mammalian cells. It is expressed in virtually all tissues, and its dysfunction is associated with various diseases such as skin blistering. There is evidence that plectin regulates the mechanical integrity of the cytoskeleton in diverse cell and tissue types. However, it is unknown how plectin modulates the mechanical response of cells depending on the frequency and amplitude of mechanical loading. Here we demonstrate the role of plectin in the viscoelastic properties of fibroblasts at small and large deformations by quantitative single-cell compression measurements. To identify the importance of plectin, we compared the mechanical properties of wild-type ($Plec^{+/+}$) fibroblasts and plectin knockout ($Plec^{-/-}$) fibroblasts. We show that plectin knockout cells are nearly twofold softer than wild-type cells, but their strain-stiffening behavior is similar. Plectin deficiency also caused faster viscoelastic stress relaxation at long times. Fluorescence recovery after photobleaching experiments indicated that this was due to threefold faster actin turnover. Short-time poroelastic relaxation was also faster in $Plec^{-/-}$ cells compared with $Plec^{+/+}$ cells, suggesting a more sparse cytoskeletal network. Confocal imaging indicated that this was due to a marked change in the architecture of the vimentin network, from a fine meshwork in wild-type cells to a bundled network in the plectin knockout cells. Our findings therefore indicate that plectin is an important regulator of the organization and viscoelastic properties of the cytoskeleton in fibroblasts. Our findings emphasize that mechanical integration of the different cytoskeletal networks present in cells is important for regulating the versatile mechanical properties of cells.

SIGNIFICANCE Mammalian cells combine superior mechanical strength with the ability to actively deform themselves. They owe this paradoxical mechanical behavior to their cytoskeleton, an intracellular web of protein filaments that includes actin filaments and intermediate filaments. It is known that both cytoskeletal filament types contribute to cell stiffness on their own, but the impact of their mechanical integration via cytoskeletal cross-linker proteins remains unknown. Here, we test the effect of cross-linking of actin and vimentin intermediate filaments by the cross-linker protein plectin in fibroblasts by single-cell compression measurements. By comparing normal cells and cells in which plectin is knocked out, we find that plectin significantly increases cell stiffness and provides a protective mechanism against actin network disruption by compressive loading.

INTRODUCTION

Cells possess an extraordinary capacity to preserve their structural integrity in physically stressful environments. In mammalian cells, the main structure governing mechanical protection is the cytoskeleton, a biopolymer network that extends throughout the cell's interior (1). The cytoskeleton is composed of four distinct biopolymers: actin, microtubules, intermediate filaments, and septins, each playing specific roles in different cellular functions (2). The filaments differ in their mechanical and dynamical properties. With respect to mechanics, intermediate filaments and septins are the softest, with a persistence length of only ~1 μm (3,4), whereas actin filaments and microtubules are stiffer, with persistence lengths of ~10 μm and several millimeters,

Submitted May 28, 2025, and accepted for publication September 2, 2025.

*Correspondence: g.h.koenderink@tudelft.nl

Mathilde G. Lettinga's present address is Center for Molecular and Cellular Bioengineering, BIOTEC, TU Dresden, Germany.

James P. Conboy and Mathilde G. Lettinga contributed equally to this work.

Editor: Guy Genin.

https://doi.org/10.1016/j.bpj.2025.09.001

© 2025 The Author(s). Published by Elsevier Inc. on behalf of Biophysical Society.
This is an open access article under the CC BY license (http://creativecommons.org/licenses/by/4.0/).

respectively (5). With respect to dynamics, actin filaments and microtubules have high polymerization and depolymerization rates, whereas intermediate filaments and septins form more stable polymers (2). Each cytoskeletal filament is organized into higher-order structures such as networks or bundles by their own dedicated set of accessory proteins such as cross-linker proteins and polymerization factors (6–9). Cell-free reconstitution experiments and theoretical models have shown that cross-linker proteins sensitively control the elastic properties of cytoskeletal networks, including the elastic modulus, rupture strength, and ability to strain-stiffen (10). In addition, the dynamic binding and unbinding of actin cross-linkers introduces time-dependent viscoelastic properties (11–13).

The mechanical functions of the different cytoskeletal filament systems have traditionally been studied separately. Recently, however, there has been growing appreciation for the importance of physical and biochemical coupling of the filaments in the ability of cells to resist, transmit, and generate mechanical forces (2). Spectraplakin proteins play a particularly important role in mechanical integration of the cytoskeleton by forming cross-links between different filament types (14,15). Spektraplakins are large (>500 kDa) and evolutionarily conserved cross-linker proteins that are part of the spectrin superfamily. Plectin is a widely expressed member of this protein family that serves as a cross-linker between actin and intermediate filaments (16,17). Human plectin is encoded by a single gene located on chromosome 8 (18), but alternative splicing creates 12 different plectin isoforms expressed in different combinations in different cell and tissue types (19).

All plectin isoforms have a C-terminal intermediate filament-binding domain and an actin-binding domain (ABD) located close to their N termini. Moreover, they all share a spectrin repeat-containing plakin domain and a central alpha-helical rod domain that mediates plectin dimerization. By contrast, the N-terminal head domain is variable. This domain controls the cellular localization of each plectin isoform by docking to distinct interaction partners such as the nucleus (plectin isoform P1), junctional complexes like focal adhesions (isoform P1f), or microtubules (isoform P1c) (19). As a consequence, plectins simultaneously cross-link intermediate filaments to actin filaments and connect these cytoskeletal networks to microtubules, the nucleus, and adhesion complexes (20). Through its connector role, plectin is essential for a stable cytoarchitecture and mechanical integrity of epithelial and endothelial tissues (21–24). Accordingly, plectin mutations result in multiple multisystemic diseases known as plectinopathies (25–27). A well-known example is epidermolysis bullosa simplex (EBS-MD), a disease characterized by spontaneous skin blistering, impaired wound healing, and muscular dystrophy (28). At the cell level, dermal fibroblasts from EBS-MD patients exhibit an anomalous vimentin network architecture, with vimentin intermediate filaments organized in bundles instead of meshworks (28). Plectin knockout in keratinocytes results in similar changes in the keratin cytoskeleton, from keratin meshworks to bundles (29).

In mesenchymal cells, plectin cross-links vimentin to different actin structures, including the actin cortex of mitotic cells (30,31), invadopodial actin networks of invading cancer cells (32), and actin stress fibers in adherent cells (33,34). One function of plectin-mediated cross-linking of actin and vimentin is to control the transmission of actin-myosin-based forces in cells, both in 2D-adherent conditions and in 3D matrices (34,35). Given this mechanical function, plectin-mediated cross-linking would also be expected to influence the stiffness of the cytoskeleton. In biochemically reconstituted actin-vimentin networks, plectin-mediated cross-linking indeed causes network stiffening (36). Surprisingly, however, measurements of plectin's contribution to cellular elasticity are contradictory. In mouse skin fibroblasts probed with magnetic twisting cytometry, plectin was found to increase cell stiffness (35). Similar behavior was observed for myoblasts probed with magnetic tweezers (37). However, this same study showed an opposite behavior for keratinocytes, where plectin knockout made cells stiffer. Atomic force microscopy (AFM) on malignant epithelial cells showed a negligible effect of plectin knockdown on cell stiffness (38). These discrepancies could point to cell-type-specific functions of plectin, caused, for instance, by different expression levels of the various plectin isoforms. Differences in the experimental assays could also contribute to the reported discrepancies. It is well known that values of cell stiffness and viscosity vary substantially depending on the level of applied mechanical stress, the rate of deformation, the geometry of the measurement probe, the location probed in the cell, and the extracellular microenvironment (39). Prior studies of the role of plectin in cell mechanics were based on local probing by AFM-based nanoindentation or magnetic tweezer manipulation of micron-sized beads attached to focal adhesions. This type of local probing has been shown to be more sensitive to cortical actin than to cytoplasmic intermediate filaments (40,41). Furthermore, these methods are limited to small deformations, whereas cells in the body tend to experience large deformations where nonlinear elastic properties become important.

Here, we investigate the mechanical role of plectin in fibroblasts at small and large deformations by single-cell compression with our newly developed device that affords high precision in strain rate and amplitude (42). To measure the whole-cell mechanical response, we confined the cells between two nonadhesive parallel plates, a deliberate design choice to isolate the intrinsic contributions of the cytoskeleton independent of adhesion-mediated effects, thus preventing any contractile active response of the cells to compression. To identify the role of plectin, we compare the linear viscoelastic properties of wild-type versus plectin knockout cells in response to small-amplitude oscillatory

and step strains. We find that plectin has a significant impact on bulk cell stiffness and also influences the time-dependent poroelastic and viscoelastic behavior of the cells. Strain ramp experiments compressing the cells into the nonlinear elastic regime confirm that the plectin knockout cells are softer than wild-type cells at small strains but show similar strain-stiffening behavior. Finally, under cyclical straining, we observed that wild-type cells are initially stiffer than plectin knockout cells but attain a similar stiffness as plectin knockout cells upon repeated loading, suggesting that plectin shields the cytoskeleton from damage by providing transient cross-links that dissociate under load.

MATERIALS AND METHODS

Cell culture and sample preparation for compression experiments

All experiments were performed with immortalized mouse fibroblast cell lines derived from transgenic plectin wild-type ($Plec^{+/+}$) mice or plectin-deficient ($Plec^{-/-}$) mice in which the plectin gene had been targeted and inactivated by homologous recombination; consequently, $Plec^{-/-}$ fibroblasts used in this study were plectin null (i.e., lacking all isoforms of plectin). Generation of transgenic mouse lines and isolation of immortalized cell lines have previously been described in detail (see (43–45)). Cells were cultured in Dulbecco's modified Eagle's medium with glutamax (DMEM, 10565018 Gibco) supplemented with 10% fetal bovine serum (FBS, 10270106 Gibco) and 5% penicillin-streptomycin (Pen-Strep antibiotic, 15070063 Gibco). Cells were maintained by subculturing twice a week and were screened for mycoplasma contamination every 4 months. Experiments were performed with cells up to passage number 20.

One day before experiments, cells were detached with 0.25% Trypsin-EDTA (25200056 Thermo Fisher Scientific) and counted using a Countess cell counter (Thermo Fisher Scientific). Next, 10,000 cells were transferred to plastic cell culture six-well plates (83.3920.005 Sarstedt) containing culture medium. The cells were left to grow to ~70% confluence. On the day of the experiments, the cells were stained by replacing the medium with medium containing CellTracker Orange (10082742 Thermo Fisher Scientific, 1:1000 dilution). After 30 min incubation, the medium was removed, and the cells were detached from the plate by incubating with 200 µl Trypsin-EDTA (0.25%) for 3 min. The cells were then suspended in 4 mL CO_2-independent medium (18045088 Gibco), prewarmed to 37°C. Next, the cells were seeded into 35-mm homemade dishes. Dishes were made by glueing 19-mm glass coverslips (12323138 Thermo Fisher Scientific) precleaned by sonication in 2-propanol (5 min) to Petri dishes (Sarstedt) with Norland Optical Adhesive glue. A drop of glue was placed in the dish, the coverslip was carefully placed on top to make sure the glue was evenly spread, and the glue was cured for 5 min using a UV ozone ProCleaner (Bioforce Nanosciences). The dishes were coated with Pluronic F-127 (P2443 Sigma) dissolved (1:100 w/v) in phosphate-buffered saline (PBS) (10010023 Gibco) for 15 min before the experiment to prevent cell adhesion.

Single-cell compression experiments

Cell compression experiments were performed using a Chiaro Nanoindenter operated by the Piuma software (Optics11 Life) mounted on a Leica Thunder Imager widefield microscope. The cells were imaged in situ using a 200-mW solid-state LED5 light source (Leica), a monochrome sCMOS camera (Leica), and a 10× dry objective (HC PL APO 0.45NA, Leica). We used custom-made wedged cantilevers prepared by modification of tipless cantilevers with a nominal spring constant of 0.018 N/m (Optics11 Life). To correct for the 3° tilt of the cantilevers (46), we fabricated a wedge using Norland optical adhesive (NOA81 Norland), as described in our previous work (42). We calibrated the cantilevers before and after wedge manufacture by the stiff-surface contact method (as described by the manufacturer) to verify that the spring constant was unchanged.

In preparation for compression experiments, a cell dish containing 4 mL of prewarmed CO_2-independent medium was placed on the microscope stage. Before lowering the cantilever probe into the medium, we added a drop of medium onto the probe to prevent air bubbles. The probe was first calibrated by the stiff-surface contact method, which involves pressing against the coverslip. Next, the cantilever was moved up, and 200 µl of cell suspension was added to the dish. After allowing cells to sediment for 10 min, the cantilever was moved down to a distance of ~200 µm above the coverslip. We then positioned a single cell underneath the cantilever with help of the fluorescent signal of the CellTracker dye in the cytosol. We carefully lowered the cantilever until reaching contact with the cell, apparent from the optically detected cantilever deflection. In all experiments, we controlled the indentation (Indentation mode in the Piuma software), which allows precise control of the rate of deformation. During measurements, the elapsed time, piezo displacement, and cantilever deflection were recorded at a time resolution of 1000 Hz. We verified by live-dead staining that the cells remained viable over the entire duration (at most 120 min) of the experiments (42).

We used three different protocols to deform the cells, selecting strain rates to reflect physiologically relevant mechanical cues experienced by fibroblasts in vivo. Fibroblasts in connective tissues are routinely subjected to mechanical strains of varying magnitudes and rates due to body motion, muscle contraction, and organ-level dynamics (47). For instance, dermal fibroblasts in the breast region near the heart can experience cyclic stretch at 1 Hz during cardiac pulsation, whereas ligament fibroblasts respond to slower, rhythmic loading around 0.1 Hz (48,49). First, for small-amplitude oscillatory measurements, we lowered the cantilever probe by 1 µm after finding the cell surface, corresponding to a compressive strain of ~0.05, which is well within the linear elastic regime. Next, a sinusoidal displacement with an amplitude of 200 nm was applied at five logarithmically spaced frequencies between 0.1 Hz and 10 Hz, corresponding to strain rates between 0.02 µm/s and 2 µm/s. Second, for stress relaxation measurement, we applied a small step strain by quickly compressing the cell by a distance of 1 µm (corresponding to a strain of ~0.05) at a large strain rate of 10 µm/s. We subsequently measured the time-dependent relaxation of the stress over a period of 80 s. Third, to capture the nonlinear response of the cells to high levels of compressive strain, we performed strain ramp measurements at a constant strain rate of 1 µm/s up to a maximal deformation of 8 µm, corresponding to a strain of ~0.4.

Analysis of single-cell compression data

All data analysis was performed using custom-written Python code available upon request. The load measured by the Nanoindenter was converted to compressive engineering stress based on the initial contact area between the cell and the cantilever. The contact area was determined from automated analysis of epifluorescence images of the cytosol by thresholding the images using Otsu's method in the Python library scikit-image (50) and multiplying the number of pixels by the pixel size in the thresholded image. The uniaxial strain was calculated by approximating cells as a sphere and dividing the amount of compression by the cell diameter. We note that the measurements and all analyses were previously benchmarked using cell-sized polyacrylamide microgel particles of known stiffness (42). Curve fitting was done using the curve fit function from Python's SciPy library for oscillatory and strain ramp measurements (51) and the Julia RHEOS package (52) for stress relaxation experiments.

Small-amplitude oscillatory measurements

We calculated the complex viscoelastic modulus, $E^*(\omega) = FT(\sigma(t))/FT(\epsilon(t))$, at each oscillation frequency $\omega = 2\pi f$, from the

Fourier transformed stress, $\sigma(\omega)$, and strain, $\epsilon(\omega)$. The Fourier transformations were performed using the fast Fourier transform function in Python's numpy library. We separated the complex modulus, defined as $E^* = E' + iE''$, into its real and imaginary parts to obtain the compressive storage modulus, $E'(\omega)$, and loss modulus, $E''(\omega)$. $E'(\omega)$ corresponds to the elastic modulus of the cell (i.e., the energy stored per cycle of oscillation), whereas $E''(\omega)$ corresponds to the viscous component (i.e., the energy dissipated per cycle of oscillation).

To mechanically fingerprint our cells, we first tried to fit the frequency spectra to the simplest possible phenomenological model referred to as the structural damping model (53–55) (for more details see supporting material). Briefly, this three-parameter model is described by the following:

$$E^*(\omega) = E_0 c(\alpha)(1 + i\tan(\pi\alpha/2))(\omega/\omega_0)^\alpha + i\mu\omega, \quad (1)$$

where E_0 is a scaling factor for the elastic and viscous moduli at a frequency scale factor ω_0 (which we arbitrarily set to 1), $c(\alpha) = \Gamma(1-\alpha)\cos(\pi\alpha/2)$ with Γ the gamma function, α is the power law exponent, and μ is the Newtonian viscous term. As this model failed to capture the rheology of our cells, we applied a fractional rheological model adapted from recent work of Bonfanti et al. (55,56):

$$E^*(\omega) = k\frac{\left[i\omega\frac{\eta_1}{k} + 1 + \left(i\omega\frac{c_\beta}{\eta_1}\right)^{1-\beta}\right]}{1 + \left(i\omega\frac{c_\beta}{\eta_1}\right)^{1-\beta}} + i\omega\eta_2 \quad (2)$$

This model has five fitting parameters: k is the elastic modulus representing the stiffness of the cell, η_1 is a dashpot viscosity capturing long-term viscous dissipation, c_β is a spring-pot parameter describing the intermediate viscoelastic behavior, β is a fractional exponent that characterizes power law scaling with frequency, and η_2 is a Newtonian viscous term capturing the response of the cytoplasm. We further decomposed Eq. (2) into real and imaginary parts:

$$E'(\omega) = k + \frac{\omega\,\eta_1\cos\left(\beta\frac{\pi}{2}\right)}{(\omega\tau)^{-(1-\beta)} + 2\sin\left(\beta\frac{\pi}{2}\right) + (\omega\tau)^{1-\beta}}, \quad (3)$$

$$E''(\omega) = \frac{\omega\,\eta_1\left[1 + (\omega\tau)^{1-\beta}\sin\left(\beta\frac{\pi}{2}\right)\right]}{1 + 2(\omega\tau)^{1-\beta}\sin\left(\beta\frac{\pi}{2}\right) + (\omega\tau)^{2(1-\beta)}} + \omega\,\eta_2, \quad (4)$$

For both models, we simultaneously fitted the measured storage and loss moduli to these equations to find a self-consistent description of the elastic and viscous response.

Step strain measurements

We found that stress relaxation after a step strain exhibited two distinct time regimes characterized by different functional forms. In the first regime, for times between 10^{-3} and 10^{-1} s, we fitted the stress response to a poroelastic model, where stress decays exponentially with time (57):

$$E(t) = E_0 e^{-t/\tau_p} + E_\infty. \quad (5)$$

Here, E_0 denotes the instantaneous modulus immediately after loading, E_∞ is the equilibrium modulus at long times, and τ_p is the poroelastic relaxation time. In the second regime (times between 0.1 and 80 s), where the cell behaves as a viscoelastic material, we fitted the same five-parameter fractional rheological model as with the oscillatory measurements. To obtain the relaxation modulus $E(t)$, we calculated the Laplace transform of Eq. (2), which yields

$$E(t) = c_\beta t^{-\beta} E_{1-\beta,1-\beta}\left(-\frac{c_\beta}{\eta_1}t^{1-\beta}\right) + k, \quad (6)$$

where $E_{a,b}(z)$ denotes the Mittag-Leffler function, a function that naturally appears in the solutions of fractional differential equations (39). To obtain parameter fits, we fit Eq. (6) to the stress relaxation data using the Julia RHEOS package (52).

Strain ramp measurements

The differential cell stiffness K was calculated as the derivative of the stress versus strain curves using the Python np.gradient function (58). The linear modulus K_0 and the onset strain (ϵ^*) and onset stress (σ^*) where stress-stiffening sets in were determined by performing a piecewise linear fit to the stress/stiffness curve for each individual cell. We fitted a horizontal line and a linear equation of the form $y = mx + c$ with the Python scipy library (51), optimizing the onset point by maximizing the combined R^2 values of both fits. The linear modulus was given by the y-intercept of the horizontal line. To rescale the stress/stiffness curves onto a single master curve, we rescaled K by K_0 and σ by σ^*.

Confocal microscopy experiments

Confocal imaging of the cells was performed on a Leica Stellaris 8 LSCM microscope, using a 63×, NA 1.30 glycerol immersion objective, white light laser, and HyD S and HyD X detectors in counting mode. Four days before experiments, the vimentin cytoskeleton was labeled by transfecting the cells with a GFP-vimentin plasmid (59) using electroporation with the Invitrogen Neon transfection system (Thermo Fisher Scientific) according to the manufacturer's instructions. A single pulse of 1350 V was applied for 30 ms. We found that cells required 4 days to express vimentin at stable levels. For actin labeling, on the day of experiments, cell culture medium was replaced with fresh medium containing SiR actin (Tebu Bio, SC001) (1:1000 from a 1 mM stock solution) along with the pump inhibitor verampamil (Tebu Bio, SC001, part of SiR actin kit) (1:1000 from a 1 mM stock solution). After a 1-h incubation, the medium was replaced with fresh medium containing Hoechst 33342 (Thermo Fisher Scientific, H3570) (1:10,000) to label the nucleus. The dye incubation was done directly before imaging. To mimic the conditions of the single-cell compression experiments, cells were trapped between two coverslips coated with Pluronic F-127 (Sigma, P2443) spaced apart by 10-μm-sized silica particles (Sigma 904368). We acquired 3D confocal z-stacks of 256 × 256 pixel xy-slices separated by a z-distance of 0.33 μm at a scan speed of 400 Hz.

Analysis of confocal microscopy data

The orthogonal projection tool in FIJI (60) was used to generate side projections of cells. To generate "average cells," a maximum intensity projection was generated from a 2-μm section centered at the cell equator. Multiple-channel stacks (containing projections of the actin, vimentin, and the nucleus) were aligned using the HyperStackReg plugin (61), which registered cells on top of each other, correcting for differences in cell size. In FIJI, we then made average intensity projections to generate an average cell. Line profiles of the actin, vimentin, and nucleus signals were determined both for single cells and "average cells." To this end, the radial profile plot tool in FIJI was used to calculate the normalized intensities around concentric circles from the center of the image. Images of at least 12 cells were collected from at least two independent experiments for each condition.

FRAP measurements of cytoplasmic viscosity and actin network turnover

Fluorescence recovery after photobleaching (FRAP) measurements were performed using the same Leica Stellaris 8 confocal microscope described above, operated in FRAP mode in the LASX software. To ensure effective photobleaching, we used the *zoom-in* and *background to zero* settings as well as the FRAP booster in the Leica LASX software. Cells were again trapped between two coverslips spaced apart by 10-μm-sized beads to mimic the geometry of the compression experiments. For determining cytoplasmic viscosity, the cells were stained with CellTracker Orange (10082742 Thermo Fisher Scientific, 1:1000 dilution in medium) on the day of the experiments. For determining actin turnover, the actin cytoskeleton was labeled by transfecting the cells with an actin-GFP plasmid (62) using electroporation with the Invitrogen Neon transfection system (Thermo Fisher Scientific). A single pulse of 1350 V was applied for 30 ms.

For both CellTracker and actin FRAP experiments, we imaged a 128 × 128 pixel confocal xy-slice using the HyD S detector. First, two prebleach images were taken to determine the fluorescence intensity before bleaching. Both the CellTracker dye and the actin-GPF were photobleached within a circular region with a radius of 1 μm in the central xy-plane of the cell. This diameter was chosen to ensure that recovery is clearly visible in the images, while minimizing cell damage, and so that we could sample the recovery with sufficient time resolution (62). To achieve efficient bleaching, we performed two exposures, each lasting 0.23 s, using four laser lines operated at 100% intensity (531 nm, 541 nm, 551 nm, and 561 nm for CellTracker; 458 nm, 468 nm, 478 nm, and 488 nm for actin-GFP). After photobleaching, we recorded 200 postbleach images every 0.23 s for both CellTracker and actin-GFP. For cytoplasm recovery measurements on $Plec^{+/+}$ cells, we performed 38 measurements in 11 different cells, and 36 measurements on 11 cells for $Plec^{-/-}$ cells. For actin turnover measurements, we performed 35 measurements in 15 different $Plec^{+/+}$ cells and 27 measurements in 14 different $Plec^{-/-}$ cells. Measurements were performed at varying locations distant from the nucleus and the cell membrane.

Analysis of FRAP measurements

To determine the fluorescence recovery times for CellTracker and actin-GFP after photobleaching, we analyzed the time-dependent intensity during recovery $I(t)$ in the bleached region throughout fluorescence recovery using a custom-written Python code. We computed the normalized intensity $I_n(t)$ by dividing $I(t)$ by the prebleach intensity I_i. We corrected for bleaching by the probe beam by also measuring the fluorescence over time in a reference region far away from the bleaching region. Assuming a two-dimensional circular region of interest and a uniform laser disk profile, we determined the FRAP recovery time by fitting $I_n(t)$ to the following model (63):

$$I_n = \exp\left(-\frac{2\tau}{t}\right)\left[\mathbf{I}_0\left(\frac{2\tau}{t}\right)+\mathbf{I}_1\left(\frac{2\tau}{t}\right)\right]+I_\infty, \qquad (7)$$

where τ is the characteristic recovery time, $\mathbf{I}_0, \mathbf{I}_1$ are modified Bessel functions, and $I_\infty < 1$ is the intensity asymptote due to an immobile fraction within the sample. For CellTracker, we can infer a diffusivity D from the relationship $\tau = r^2/(4D)$, with r being the bleach radius (63). It reacts with glutathione, which is most abundant in the cytoplasm but is also present in subcellular organelles such as mitochondria (64). We thus expect to see partial rather than total fluorescence recovery (i.e., $I_\infty > 0$). In the case of actin-GFP, we expect full fluorescence recovery (62).

Western blotting

Cells were seeded in six-well culture plates (Thermo Fisher Scientific, NC0506188) containing 2 mL of culture medium and allowed to attach for 48 h. Cells were scraped in PBS and lysed in equal volumes of 2× Laemmli buffer (4% sodium dodecyl sulfate (SDS), 20% glycerol, 120 mM tris(hydroxymethyl)aminomethane buffer (pH 6.8)). Lysates were cleared of large DNA by passing through a 25G needle and then heated to 65°C for 10 min. Protein concentrations were measured with the Lowry protein assay (65). Equal amounts of protein were size-separated by SDS-PAGE containing 2,2,2-trichloroethanol. After running the gel, it was exposed to a 300-nm transilluminator (Bio-Rad ChemiDoc Imaging System) for 5 min. The separated proteins were then transferred to a polyvinylidene fluoride membrane (Bio-Rad, 1704156) using the Bio-Rad Trans-Blot Turbo Transfer System and imaged on the transilluminator to get a picture of the total protein. Membranes were blocked for 1 h at room temperature by 5% BSA in PBS supplemented with 0.1% Tween-20 and incubated overnight at 4°C with primary antibody (see Table S2). Subsequently, membranes were washed five times with 0.1% Tween-20 in PBS and then incubated with horseradish peroxidase-conjugated secondary antibodies (see Table S2) for 1 h at room temperature. After adding enhanced chemiluminescence substrate (Thermo Fisher Scientific, 32106), blots were imaged with the Bio-Rad ChemiDoc Imaging System. The band intensities were quantified using FIJI (60).

Statistics

All mechanical experiments shown were repeated on at least 3 different days. All imaging (including FRAP) experiments were repeated on 3 different days. Measured values in the main text are stated as the mean ± the standard error on the mean. For all mechanical measurements, p-values to quantify statistical significance were calculated using the Wilcoxon rank-sum test after assessing normality with the Shapiro-Wilk test, which confirmed that the data did not follow a Gaussian distribution. For FRAP measurements, p-values were calculated using the unpaired Student's t-test. Computer codes described in the materials and methods section are available on request.

RESULTS

Plectin influences actin and vimentin organization in fibroblasts

The aim of this study was to determine the contribution of plectin to the viscoelastic behavior of fibroblasts at small and large deformations. To this end, we made a side-by-side comparison between immortalized mouse fibroblasts derived from transgenic plectin wild-type ($Plec^{+/+}$) and plectin-deficient ($Plec^{-/-}$) mice (for details, see (43–45)). Western blot analysis confirmed that the $Plec^{-/-}$ cells lack plectin, whereas the expression levels of actin and vimentin are comparable to wild-type cells (Figs. 1 *C* and S1). To measure the whole-cell mechanical response, we confined the cells between two parallel plates that were made nonadhesive to prevent any active response of the cells to compression and thus isolate the intrinsic mechanical response of the cytoskeleton and nucleus.

To characterize the impact of plectin knockout on the organization of the cytoskeleton in this geometry, we first imaged cells confined between two nonadhesive coverslips spaced apart by a distance of 10 μm (Fig. 1 *A*). This separation distance is smaller than the average cell size determined by imaging flow cytometry measurements (Fig. S2), thus allowing us to image our cells in a compressed state. $Plec^{+/+}$ and $Plec^{-/-}$ cells had comparable diameters (22.4 ± 3.1 μm and 22.2 ± 3.1 μm, respectively; see Fig. S2 *A*) and nucleus

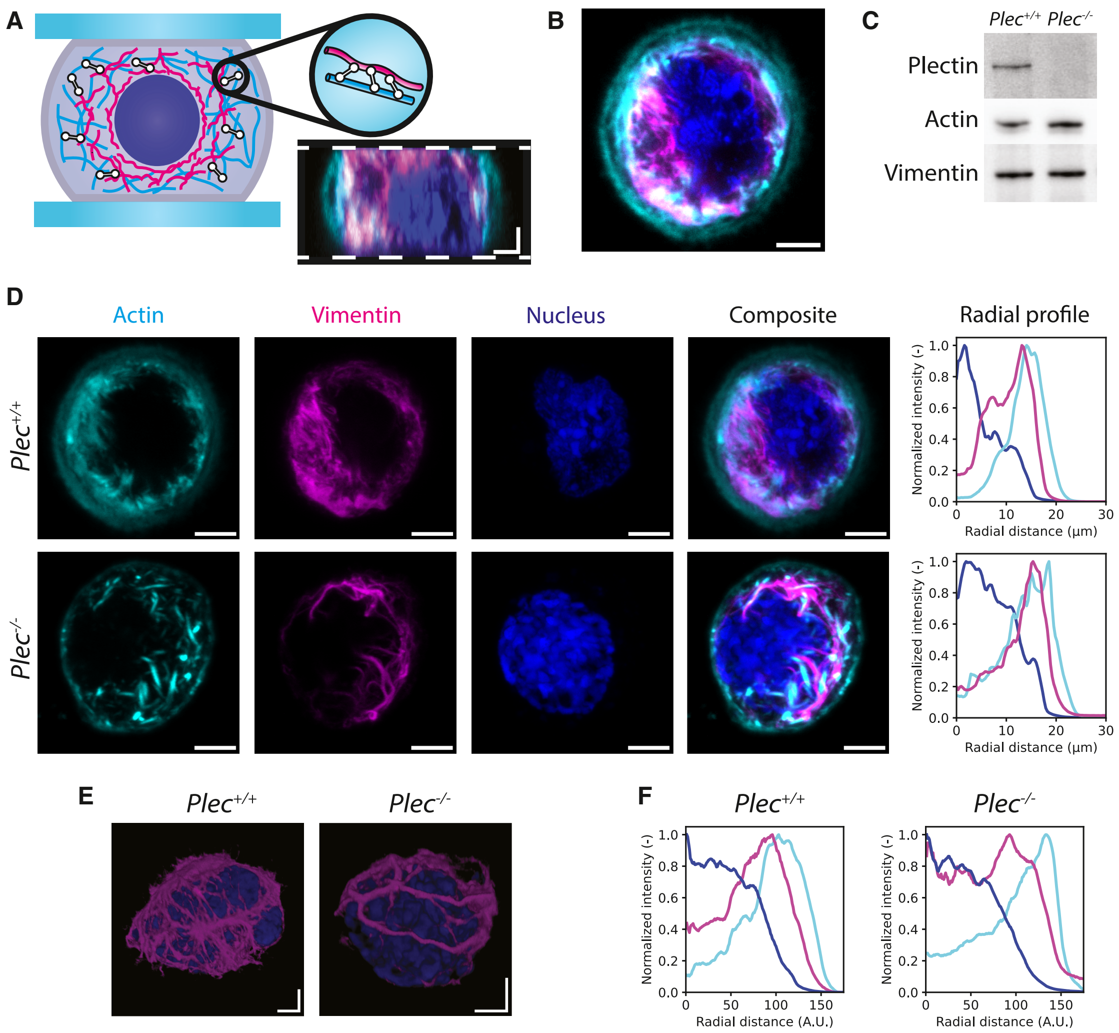

FIGURE 1 Plectin influences the organization of the actin and vimentin cytoskeleton in nonadherent fibroblasts. (*A*) The schematic shows how live fibroblasts were imaged while trapped between two nonadhesive surfaces. Plectin (*white*) forms cross-links between actin filaments (*cyan*) and vimentin intermediate filaments (*magenta*). The image shows a side projection of a *Plec*$^{+/+}$ fibroblast. White dashed lines indicate the two confining surfaces. Scale bars represent 3 µm. (*B*) Single confocal slice through the cell equator of a *Plec*$^{+/+}$ fibroblast. SiR-labeled actin (*cyan*) is concentrated at the cortex, whereas vimentin-GFP (*magenta*) surrounds the nucleus (*dark blue*). Scale bar, 3 µm. (*C*) Immunoblot analysis of cell lysates from *Plec*$^{+/+}$ and *Plec*$^{-/-}$ cells using antibodies to plectin, actin, and vimentin. (*D*) Representative images of *Plec*$^{+/+}$ and *Plec*$^{-/-}$ cells. Images are maximum intensity projections of 2-µm-thick confocal sections at the cell equator. Actin is located largely at the cell periphery, whereas vimentin is located closer to the nucleus. Average radial profiles of the fluorescent signals (*right*) were calculated by taking normalized intensities around concentric circles from the center of the image. The radial distance is set to zero at the cell center. We observe a significant overlapping region between actin and vimentin. (*E*) 3D volume renderings of vimentin and the nucleus. In the *Plec*$^{+/+}$ cell, a cage-like vimentin structure surrounds the nucleus, whereas in the *Plec*$^{-/-}$ cell, vimentin forms bundles that no longer surround the nucleus on all sides. (*F*) We repeat the radial profile procedure shown in (*D*) by averaging the images of multiple cells ($N = 10$ for both conditions) and then computing the radial average. Average profiles resembled those of individual cells. Note that the scale of the "average cell" images and line profile plots are in arbitrary units since the cells were rescaled to compensate for variations in cell size.

sizes (8.3 ± 1.4 µm and 8.2 ± 1.4 µm, respectively; see Fig. S2 *B*). Side projections (Fig. 1 *A*) and top views (Fig. 1 *B*) reconstructed from confocal z-stacks showed that the cells are quasi-spherical with a radially rather uniform distribution of both actin and vimentin. Actin predominantly lines the plasma membrane at the cell periphery but also extends into the cell interior (Fig. 1 *D*). By contrast, vimentin is primarily distributed between the actin-rich region and the nucleus (Fig. 1 *D*). Most strikingly, the vimentin network in the *Plec*$^{-/-}$ cells displays increased bundling compared with the finer vimentin meshwork in *Plec*$^{+/+}$ cells (second column in Fig. 1 *D*, compare top and bottom rows). This effect is strongly reminiscent of earlier observations of vimentin filament bundling in EBS-MD patient cells (28) and of keratin filament bundling in *Plec*$^{-/-}$ keratinocytes (29). Furthermore, three-dimensional reconstructions of confocal z-stacks reveal that vimentin filaments in *Plec*$^{+/+}$ cells are positioned in close proximity to the nuclear envelope,

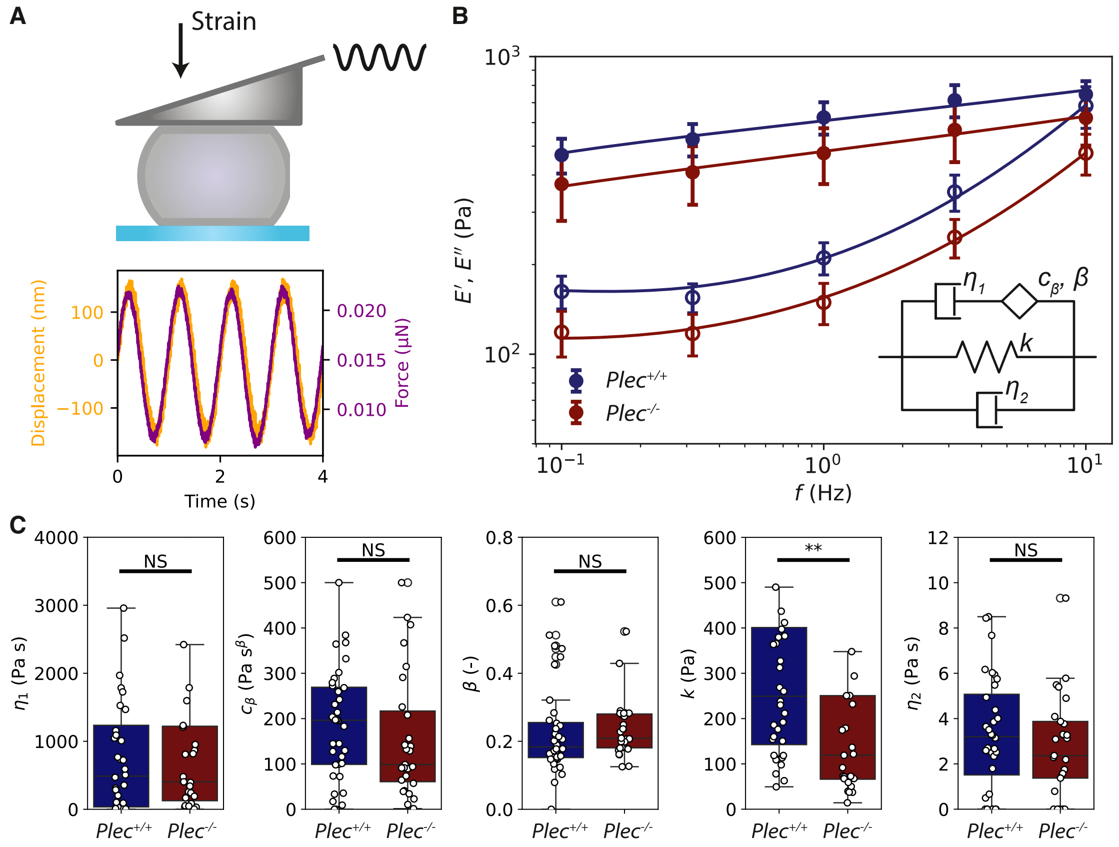

FIGURE 2 Small-strain oscillatory compression measurements on single fibroblasts reveal that $Plec^{+/+}$ cells are stiffer than $Plec^{-/-}$ cells. (*A*) Top: illustration depicting a single cell positioned between a glass coverslip and the wedged cantilever. Bottom: typical measurement for a $Plec^{+/+}$ cell at an oscillation frequency of 1 Hz, showing the cantilever's applied displacement (*black; left y-axis*) and the corresponding observed force (*red; right y-axis*) over time. (*B*) Storage (E') and loss (E'') linear compressive moduli (*solid and open symbols, respectively*), measured for oscillation frequencies between 0.1 Hz and 10 Hz for $Plec^{+/+}$ cells ($N = 36$, *blue*) and $Plec^{-/-}$ cells ($N = 27$, *red*). Fits on the average data (*solid lines*) to the fractional rheological model (*inset*) from Eq. (2). (*C*) Material parameters determined from the fits of the fractional rheological model for each cell individually. The five fitting parameters are the dashpot viscosity η_1 capturing long-term viscous dissipation, the spring-pot parameter c_β describing the intermediate viscoelastic behavior, the fractional exponent β characterizing power law scaling with frequency, the elastic modulus k representing the stiffness of the material, and the Newtonian viscous term η_2 capturing the response of the cytoplasm. Only the elastic parameter k changes upon plectin knockout, whereas all other parameters are unchanged. Asterisks indicate statistically significant differences (** denotes $p < 0.01$), and N.S. denotes nonsignificant differences.

forming a filamentous network that encases the nucleus (Fig. 1 *E*, left). This structure has been referred to as the vimentin nuclear cage (66) and has been shown to protect the nucleus from mechanical stress (67). By contrast, the thicker vimentin bundles in $Plec^{-/-}$ cells no longer form such a protective meshwork structure around the nucleus (Fig. 1 *E*, right).

To better compare the spatial distributions of actin and vimentin within the cell interior, we calculated radial distributions of the intensities. For individual cells, this showed a considerable overlap between the actin and vimentin localization (Fig. 1 *D*, right). To analyze this for many cells, we constructed "average cells" by computing the average of maximum intensity projections from 2-μm-thick confocal slices centered at the cell equator, adjusting for variations in cell size (Fig. S3). This analysis revealed similar nuclear distributions between $Plec^{+/+}$ and $Plec^{-/-}$ cells and confirmed a significant region of overlap between the actin and vimentin networks (Fig. 1 *F*). This observation suggests that the two networks interpenetrate. We observed subtle differences between $Plec^{+/+}$ and $Plec^{-/-}$ cells. The vimentin network on average extended to the cell periphery in $Plec^{-/-}$ cells but not in $Plec^{+/+}$ cells. The peak intensity for actin was close to the peak intensity for vimentin in $Plec^{+/+}$ cells but closer to the cell periphery in $Plec^{-/-}$ cells. We conclude that plectin has a significant impact on the organization of the actin and especially the vimentin cytoskeleton.

Small-amplitude oscillatory compression shows that plectin stiffens cells

To investigate how plectin removal impacts the mechanical response of the cells to small deformations, we used a single-cell compression device equipped with a wedged flexible cantilever. We subjected the cells to an oscillatory

compressive strain with a small-amplitude (∼1%) and stepwise increasing oscillation frequency from 0.1 Hz to 10 Hz (Fig. 2 *A*). From the resulting force on the cantilever, we calculated the real and imaginary parts of the frequency-dependent complex apparent Young's modulus, $E^*(f)$. The real part corresponds to the storage modulus $E'(f)$, which reflects the elastic component of the response (solid symbols in Fig. 2 *B*). The imaginary part corresponds to the loss modulus $E''(f)$, which reflects the viscous component of the response (open symbols in Fig. 2 *B*). Both moduli show a qualitatively similar dependence on frequency for the $Plec^{+/+}$ cells (blue) and $Plec^{-/-}$ cells (red). In both cases, E' is larger than E'', in particular at low frequencies, consistent with predominantly solid-like behavior. Furthermore, the elastic moduli increase with a weak power law dependence on frequency, characteristic for mammalian cells (68). At the highest frequency, the elastic and viscous moduli tend toward a crossover. In quantitative terms, however, we see a clear difference: the moduli of $Plec^{+/+}$ cells are greater than those of $Plec^{-/-}$ cells by a factor ~ 1.4, indicating that plectin stiffens the cells. Additionally, we observe a shift in the projected crossover frequency for the elastic and viscous moduli, from ∼12 Hz in $Plec^{+/+}$ cells to ∼16 Hz in $Plec^{-/-}$ cells, signifying that $Plec^{-/-}$ cells may maintain an elastic mechanical response over a broader frequency range.

For a more in-depth comparison of the $Plec^{+/+}$ and $Plec^{-/-}$ cells, we fitted the experimental data to phenomenological models that describe the rheology in terms of a small number of characteristic material parameters. We first attempted to describe the data in terms of the structural damping law, an established phenomenological model of cell rheology (53,54). This model (see Eq. (1)) has the benefit that it only has three material parameters, namely the scaling factor E_0, power law exponent α, and cytoplasmic viscosity μ. We previously found that this model could capture the compressive modulus of mouse embryonic fibroblasts rather accurately (42). However, we found that it strongly underestimated the viscous modulus of the fibroblasts studied in this work, particularly at low frequencies (Fig. S4). Apparently, we need to include an additional viscous contribution that captures slow stress relaxation. Therefore, we developed a new fractional rheological model inspired by recent work (55,56) that combines short-term power law stress relaxation and long-term exponential stress relaxation. This model (see Eq. (2)) has five free parameters: the elastic modulus k representing the stiffness of the material, the dashpot viscosity η_1 capturing long-term viscous dissipation, the spring-pot parameter c_β describing the intermediate viscoelastic behavior, the fractional exponent β characterizing power law scaling with frequency, and finally the Newtonian viscous term η_2 capturing the response of the cytoplasm. As shown in Fig. 2 *B* (solid lines), this model provides an accurate and self-consistent fit of E' and E''. The only fitting parameter that is significantly different between the $Plec^{+/+}$ and $Plec^{-/-}$ cells is the elastic modulus k, which decreases from $k = 232 \pm 20$ Pa in $Plec^{+/+}$ cells ($N = 36$) to $k = 120 \pm 20$ Pa in $Plec^{-/-}$ cells ($N = 27$) (Fig. 2 *C*). The two parameters characterizing the power law scaling behavior, β and c_β, are comparable between $Plec^{+/+}$ and $Plec^{-/-}$ cells. Likewise, the dashpot viscosity η_1 and Newtonian damping term, η_2, were unchanged. We conclude that plectin only affects the elastic properties of the cytoskeleton at small deformations, within the frequency range probed here.

Plectin affects poroelastic and viscoelastic stress relaxation

The oscillatory compression measurements suggest that there is significant energy dissipation on timescales beyond 10 s (i.e., at frequencies below 0.1 Hz), the measurement limit of our instrument. Since many organs and connective tissues experience deformations that persist for minutes (69,70), we decided to measure the viscoelastic behavior of the $Plec^{+/+}$ and $Plec^{-/-}$ cells on longer timescales via step strain experiments (Fig. 3 *A*, top). We subjected cells to a rapid compression by a distance of 1 μm, corresponding to a small (~ 0.05) strain within the linear elastic regime. We then measured the stress relaxation dynamics while holding this displacement for 80 s (Fig. 3 *A* bottom). As shown in Fig. 3 *B*, both $Plec^{+/+}$ cells (blue solid line) and $Plec^{-/-}$ cells (red solid line) display marked stress relaxation over this timescale (for data of individual cells, see Fig. S5). The stress is overall larger for the $Plec^{+/+}$ cells compared with the $Plec^{-/-}$ cells, indicating that plectin depletion causes cell softening, consistent with the small-amplitude oscillatory data. In a log-log representation, we can distinguish multiple relaxation regimes. At short times (<0.1 s, indicated by a gray background in Fig. 3 *B*), we observe an exponential stress decay. Here, the mechanical response to compression is known to be dominated by the flow of cytosol through the porous elastic structure provided by organelles and the cytoskeleton (62) (see schematic inset of Fig. 3 *B*). Beyond 0.1 s, the stress relaxation response transitions to a power law time dependence, reflecting a broad distribution of relaxation processes. In this regime, we indeed expect power law viscoelastic stress relaxation just like in the small-amplitude oscillatory measurements. This is followed by exponential relaxation at long times.

To test how plectin changes the poroelastic and viscoelastic response of the cells, we fitted the stress relaxation data in the short and intermediate time regimes to corresponding theoretical model predictions. The short-time poroelastic response is expected to be single exponential (Eq. (5)) with a characteristic poroelastic decay time τ_p, assuming that the cell can be approximated as a simple biphasic material consisting of a porous elastic meshwork

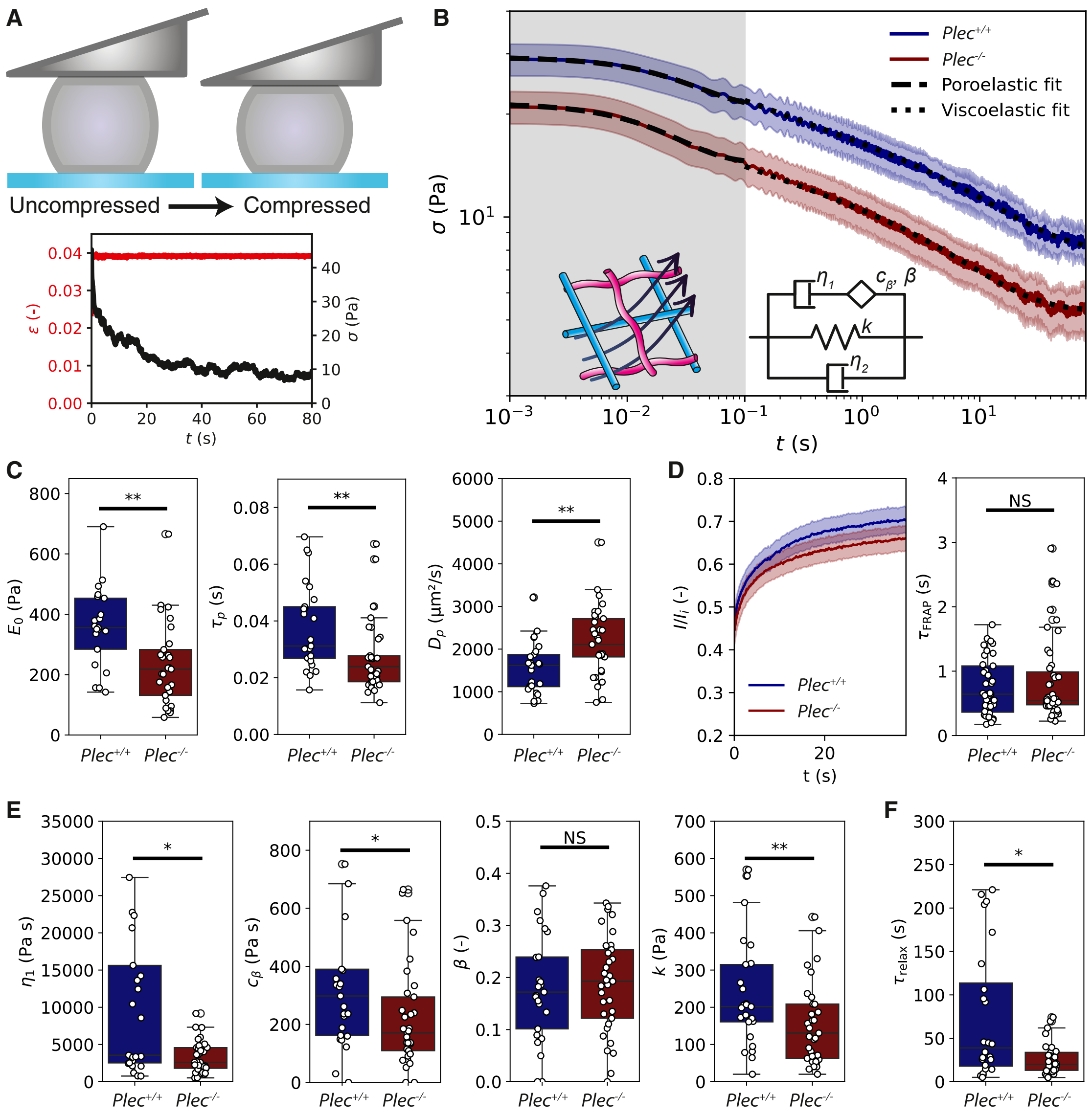

FIGURE 3 Small-amplitude stress relaxation experiments for $Plec^{+/+}$ and $Plec^{-/-}$ cells. (*A*) Top: schematic of the step strain experiment (not to scale; the strain is only 0.05). Bottom: typical measurement for a $Plec^{+/+}$ cell showing the cantilever displacement that is held constant (*red; left y-axis*), whereas the force (*black; right y-axis*) is measured over time. (*B*) Averaged stresses for $Plec^{+/+}$ cells (*blue solid line*, $N = 25$) and $Plec^{-/-}$ cells (*red solid line*, $N = 33$) over time in log-log representation. The shaded areas around the average relaxation curves represent the standard deviation. Stresses are higher for $Plec^{+/+}$ cells, indicating these cells are stiffer. At short timescales (from 0.001 s to 1 s, indicated by the gray background), the cells display exponential stress relaxation characteristic of a poroelastic material, where fluid flows through the cytoskeletal network (*inset drawing*). Fits to the poroelastic model (Eq. (5)) are shown as dashed lines. At later times (from 0.1 s to 80 s, *white background*) the data are well-described by the same fractional rheological model (*inset schematic*) that also captures the oscillatory data, transformed to the time domain (*fits shown as dotted lines*; Eq. (6)). (*C*) Elastic moduli E_0 (*left*) and poroelastic timescales τ_p (*middle*) determined from the poroelastic model fits for individual cells. The poroelastic diffusion coefficient D_p (*right*) calculated from τ_p data is faster for $Plec^{-/-}$ cells than for $Plec^{+/+}$ cells. (*D*) Comparison of cytoplasmic diffusion via fluorescence recovery after photobleaching (FRAP). Left: average FRAP recovery curves for $Plec^{+/+}$ and $Plec^{-/-}$ cells. The error band shows the standard error. Right: we measure no significant difference between the FRAP recovery times, τ_{FRAP}, of $Plec^{+/+}$ and $Plec^{-/-}$ cells. (*E*) Material parameters obtained from the fractional rheology fits, showing the dashpot viscosity η_1 describing long-term viscous dissipation, the spring-pot parameter c_β describing the intermediate viscoelastic behavior, the fractional exponent β characterizing power law scaling with frequency, and the elastic modulus k representing cell stiffness. (*F*) Viscoelastic stress relaxation timescale τ_{relax} calculated from the data in (*E*) using Eq. (10). Asterisks indicate statistically significant differences (* denotes $p < 0.05$; ** $p < 0.01$), and N.S. denotes nonsignificant differences.

with an elastic modulus, E_0, bathed in an interstitial fluid (cytosol) of viscosity μ. The data were well described by this model (dashed lines in Fig. 3 *B*). From the fits, we found that plectin knockout significantly softens the cells, with an instantaneous elastic modulus (stiffness of the cell at t $=$ 0) of $E_0 = 230 \pm 30$ Pa for $Plec^{-/-}$ cells compared with $E_0 = 360 \pm 30$ Pa for $Plec^{+/+}$ cells (Fig. 3 *C*, left). Furthermore, poroelastic relaxation occurs more rapidly upon plectin knockout, with a poroelastic timescale of $\tau_p = 0.030 \pm 0.001$ s in $Plec^{-/-}$ cells compared with $\tau_p = 0.040 \pm 0.001$ s in $Plec^{+/+}$ cells (Fig. 3 *C*, middle).

The difference in poroelastic timescales suggests that the porosity of the cytoplasm might be higher in $Plec^{-/-}$ cells compared with $Plec^{+/+}$ cells. To test this idea, we first estimated the poroelastic diffusion constant D_p from the poroelastic timescales according to

$$\tau_p \sim L^2 / D_p \tag{8}$$

For simplicity, we assume that the elastic network of cytoskeleton and organelles is homogeneous across the distance between the cell membrane and nuclear envelope, so we can define the characteristic poroelastic length scale L as the distance between the cell membrane and the nuclear envelope. We can estimate this distance from the earlier mentioned flow cytometry measurements of the cell and nucleus sizes (Fig. S2). $Plec^{+/+}$ and $Plec^{-/-}$ cells had varying sizes, but both displayed a proportional increase of cell size with nucleus size (Fig. S2 *E* and *F*), resulting in a rather constant nucleus-to-cell diameter ratio of ~ 0.74 in both cases. We therefore assume a characteristic length scale of $L = 7.1$ μm and thus estimate $D_p = (1500 \pm 100)$ μm^2/s for $Plec^{+/+}$ cells and $D_p = (2200 \pm 100)$ μm ^{2}/s for $Plec^{-/-}$ cells (Fig. 3 *C* right). Note that if we assume fluid transport also occurs in the nucleus, so L equals the cell size, we still find a larger D_p in $Plec^{-/-}$ cells (see supporting material for more details). The poroelastic diffusion constant D_p depends on the pore radius ξ of the cytoskeletal network, the cytosol viscosity μ, and the elastic modulus of the drained cytoskeletal network E according to (62):

$$D_p \sim E\xi^2 / \mu \tag{9}$$

To measure the unknown cytosol viscosity and test whether it changes upon plectin knockout, we performed FRAP on the cytoplasmic dye CellTracker Orange. We photobleached a small (1 μm) circular region and observed the recovery of the fluorescent signal (Figs. 3 *D* and S6). By fitting the individual recovery curves to Eq. (7), we recovered comparable recovery times for $Plec^{+/+}$ cells ($\tau_{FRAP} = 0.53 \pm 0.05$ s) and $Plec^{-/-}$ cells (0.50 ± 0.06 s), indicating comparable cytosol viscosities. We estimate a cytoplasmic viscosity $\mu \simeq 4$ Pa s for both $Plec^{+/+}$ and $Plec^{-/-}$ cells using the Stokes-Einstein relation for the diffusion of small molecules ($D = \frac{k_B T}{6\pi\mu R_h}$) (71), assuming a hydrodynamic radius $R_h \simeq 0.5$ nm (72) for the cytoplasmic dye. Encouragingly, this viscosity value is consistent with the cytoplasmic viscosity ($\eta_2 = 3.3 \pm 0.4$) Pa s) inferred by fitting the oscillatory compression data to the fractional rheological model (Fig. 2 *C*), lending support for this model. Plugging the poroelastic diffusion times from the step strain data and the cytosol viscosity from the FRAP data into Eq. (9), we calculate that the cytoskeletal meshwork of $Plec^{-/-}$ cells ($\xi = 7.2$ μm) has larger pores than for $Plec^{+/+}$ cells ($\xi = 4.9$ μm) by a factor of ~ 1.5. Interestingly, this is consistent with the observation that the plectin knockout cells have a more open, bundled vimentin cytoskeleton than wild-type cells (Fig. 1 *D*) while having similar concentrations of vimentin and actin (Fig. 1 *C*).

We next turned to the viscoelastic stress relaxation regime at intermediate times (0.1–80 s), which should be describable by the same fractional rheological model used to model the oscillatory measurements (inset showing a schematic of the model in Fig. 3 *B*). To test this, we converted the model from the frequency domain (Eq. (2)) to the time domain (Eq. (6)), enabling a direct comparison of the characteristic mechanical parameters. Note that the model involves four fit parameters in the time domain instead of five parameters in the frequency domain, since the fifth parameter, η_2, is reduced to a Dirac delta function after the Laplace transform. The fractional rheology model indeed fitted the force-relaxation curves well (dotted black lines in Fig. 3 *B*), returning similar conclusions as the oscillatory measurements. Again, we observed a significant decrease in the elastic modulus k, from $k = 250 \pm 30$ Pa for $Plec^{+/+}$ cells to $k = 170 \pm 30$ Pa in $Plec^{-/-}$ cells (Fig. 3 *E*), but an unchanged power law scaling exponent β. However, contrary to the oscillatory measurements, the viscous modulus η_1 determined from the stress relaxation significantly decreased upon plectin knockout, from $\eta_1 = 8000 \pm 2000$ Pa for $Plec^{+/+}$ cells to $\eta_1 = 3700 \pm 500$ Pa for $Plec^{-/-}$ cells. The stress relaxation data are likely more accurate here because we could measure up to timescales of 80 s, much longer than in oscillatory measurements. We can calculate the viscoelastic relaxation time, τ_{relax}, that marks the transition from a power law decay at intermediate times to an exponential relaxation regime at longer times inaccessible in the oscillatory measurements as follows (56):

$$\tau_{relax} = \left(\eta_1 / c_\beta\right)^{\frac{1}{1-\beta}} \tag{10}$$

This timescale indicates when the dashpot characterizing long timescale relaxation processes (beyond ~ 10 s) in the cytoskeleton begins to dominate stress relaxation (73). We observed a statistically significant decrease in this timescale, from $\tau_{relax} = 75 \pm 15$ s for $Plec^{+/+}$ cells to $\tau_{relax} = 26 \pm 4$ s

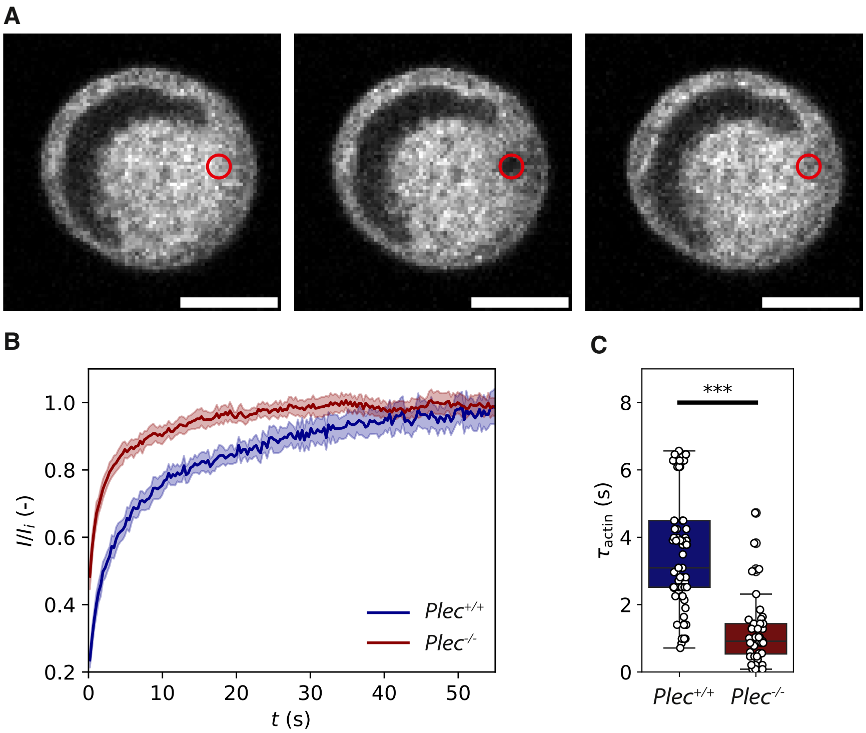

FIGURE 4 Comparison of actin turnover rates in $Plec^{+/+}$ and $Plec^{-/-}$ cells confined between two nonadhesive surfaces via fluorescence recovery after photobleaching (FRAP) experiments. (*A*) We bleach a small (1 μm) circular region (*red open circle*) in a region of the cell where we observe an actin network (tagged with GFP-actin) and monitor the fluorescence recovery over time. Note that we used a low resolution and fast imaging speed to optimize temporal sampling for accurate recovery measurements. Also note that the large dark region within the cell is occupied by the nucleus (which has a somewhat irregular 3D shape due to the parallel plate boundary conditions). Scale bar, 10 μm. (*B*) Averaged FRAP recovery curves for GFP-actin-tagged $Plec^{+/+}$ cells (*blue*, $N = 54$) and $Plec^{-/-}$ cells (*red*, $N = 35$). In both conditions, the fluorescence fully recovers, but with a significantly higher rate in the $Plec^{-/-}$ cells. (*C*) FRAP recovery times τ_{actin} obtained by fitting to the individual recovery curves (shown in Fig. S7). We find average values of $\tau_{actin} = 3.6 \pm 0.4$ s for $Plec^{+/+}$ cells and 1.4 ± 0.2 for $Plec^{-/-}$ cells, indicating about threefold faster actin turnover upon plectin knockout. *** indicates statistically significant differences ($p < 0.001$).

in $Plec^{-/-}$ cells, indicating faster stress relaxation upon plectin knockout (Fig. 3 *F*).

On long timescales, we expect that stress relaxation could occur due to unbinding of plectin and other cytoskeletal cross-linker proteins or by cytoskeletal turnover. Actin filaments turn over as a consequence of filament depolymerization, which is catalyzed by various actin-binding proteins. Actin network turnover times are typically reported to be on the order of seconds to minutes (74,75), which could correspond to the timescale where we observe stress relaxation. The vimentin cytoskeleton is typically reported to turn over on much longer timescales on the order of hours, via subunit exchange along the filaments and fragmentation (76). To determine the actin and vimentin turnover rates in our cells and test whether they are changed by plectin knockout, we performed FRAP experiments on cells tagged with GFP-actin (Fig. 4 *A*) and GFP-vimentin (Fig. S8). As shown in Fig. 4 *B*, the GFP-actin signal averaged over many cells fully recovered within 60 s for both $Plec^{+/+}$ and $Plec^{-/-}$ cells (blue and red curves, respectively; see Fig. S7 for individual curves). Strikingly, however, the actin turnover rate as quantified by the FRAP recovery time by fits to Eq. (7) was faster in $Plec^{-/-}$ cells by a factor of ~3 (Fig. 4 *C*). By contrast, FRAP experiments on GFP-vimentin transfected cells showed negligible recovery up to times of 80 s (corresponding to the timescale probed in the step strain experiments) for both $Plec^{+/+}$ and $Plec^{-/-}$ cells (Fig. S8). We therefore conclude that the faster viscoelastic stress relaxation rate in the plectin knockout cells at long times is likely due to faster actin turnover.

Plectin knockout does not impact fibroblast strain-stiffening

To test the effect of plectin on the mechanical response of fibroblasts to large compressive deformations, we performed strain ramp experiments (Fig. 5 *A*). The cells were compressed gradually with a constant rate of 1 μm/s, which is slow enough to minimize stress from viscoelastic contributions (42). Upon reaching the target deformation ($\epsilon \sim 0.4$), the cantilever was immediately retracted at the same rate. Fig. 5 *B* shows an example stress-strain cycle performed on a $Plec^{+/+}$ cell. The stress-strain curves during compression (indicated by the arrow up) and retraction (arrow down) do not overlap: for any given strain, the corresponding stress is lower during retraction than it is during compression. This hysteresis confirms the viscoelastic nature of the cells. We next compared strain ramp experiments on $Plec^{+/+}$ cells ($N = 13$) and $Plec^{-/-}$ cells ($N = 15$) (see Fig. S9 for individual stress-strain curves). The averaged data for the response upon compression reveal that $Plec^{-/-}$ cells are softer than $Plec^{+/+}$ cells: at any strain, the average resulting stress is lower for $Plec^{-/-}$ cells (red) than for $Plec^{+/+}$ cells (blue) (Fig. 5 *C*). However, the stress in both cases increases nonlinearly with the applied strain. This type of compression-stiffening behavior under uniaxial load is consistent with earlier studies (42,77).

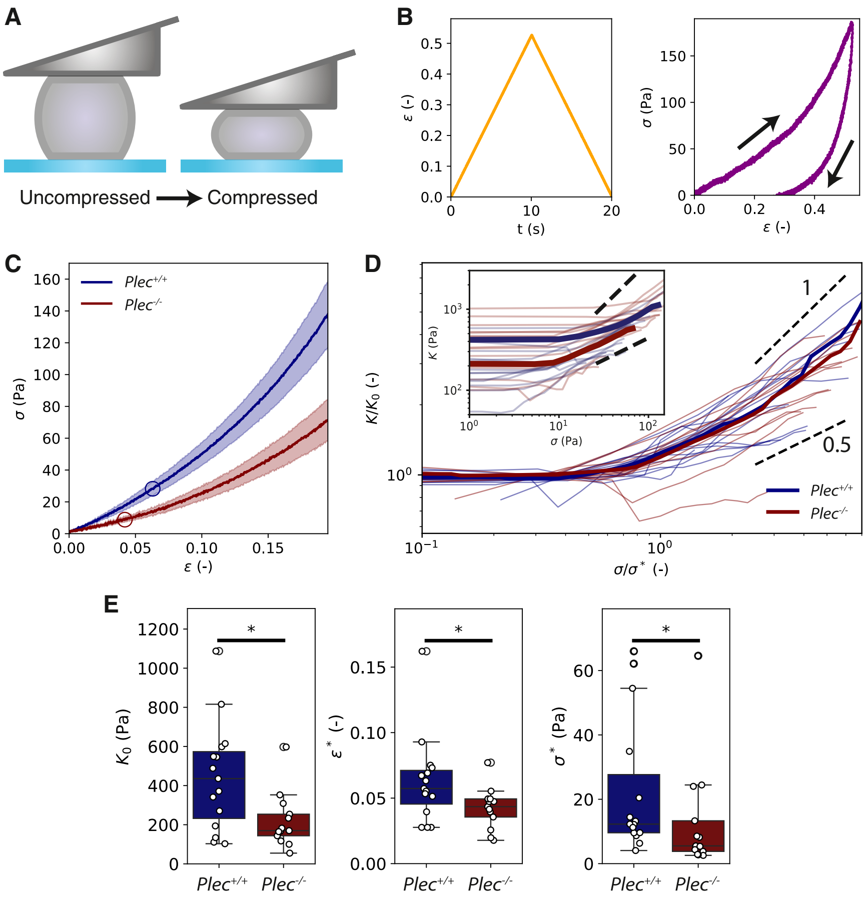

FIGURE 5 Strain ramp measurements of the nonlinear elastic response of $Plec^{+/+}$ and $Plec^{-/-}$ cells to large uniaxial compressions. (*A*) Schematic of the experiment where cells are compressed by gradually lowering the cantilever at a constant rate until a target strain of ∼0.4 and then unloaded by raising the cantilever back to its original zero-strain position. (*B*) Representative strain ramp measurement of a single $Plec^{+/+}$ cell, showing the applied strain (*left*) and concomitant stress (*right*). The arrows show the order of loading and unloading. (*C*) Average stress-strain curves for $Plec^{+/+}$ ($N = 15$) and $Plec^{-/-}$ ($N = 13$) cells showing that $Plec^{-/-}$ cells are softer than $Plec^{+/+}$ cells, but both cell types stress-stiffen. Shaded areas around the curves show the standard error. Circles indicate the onset of strain-stiffening. (*D*) Differential elastic modulus, K, for individual cells, obtained by taking the numerical derivative of the stress-strain curves from individual cells shown in Fig. S9. Inset shows the raw data; main plot shows the same data with σ normalized by the onset stress σ^* and K normalized by the linear modulus K_0. $Plec^{-/-}$ cells attain a similar stiffness as $Plec^{+/+}$ cells at large stress (*inset*), and normalization collapses the curves onto a single master curve. The short dashed lines indicate that the stress dependence falls between power law dependences with an exponent between 0.5 and 1. (*E*) Comparison of the stress-stiffening behavior of $Plec^{+/+}$ cells and $Plec^{-/-}$ cells in terms of the linear modulus, K_0 (*left*), onset strain, ϵ^* (*middle*), and onset stress, σ^* (*right*). $Plec^{-/-}$ cells (*red*) are about twofold softer than $Plec^{+/+}$ cells (*blue*) and, accordingly, display a smaller onset stress. * indicates statistically significant differences ($p > 0.05$).

To quantify this strain-stiffening behavior, we defined the differential stiffness K as $K = d\sigma/d\epsilon$. The stiffness is constant ($K = K_0$) up to a certain onset strain ϵ^*, above which stiffening occurs (Fig. 5 *D* inset). Strikingly, the stiffness at large compressive stresses of 80 Pa is comparable for $Plec^{+/+}$ cells (blue) and $Plec^{-/-}$ cells (red). Also, normalizing the stiffness by $K = K_0$ and the stress by σ^* collapses the data onto a single master curve (Fig. 5 *D*). At large deformations, the stiffness approaches a power law dependence on stress with best-fit values for the power law exponent being similar for $Plec^{+/+}$ cells (0.49 ± 0.06) and $Plec^{-/-}$ cells (0.49 ±0.05). We note that the nonlinear regime covers less than a decade in stress, so these values should be taken as indicative only. The short dashed lines in Fig. 5 *D* show that the exponents lie somewhere between 0.5 and 1.

As shown in Fig. 5 *E* (left), the linear modulus K_0 is about twofold higher for the $Plec^{+/+}$ cells (440 ±70 Pa) compared with the $Plec^{-/-}$ cells (220 ±40 Pa). This effect is consistent with the 1.7-fold difference found by small-amplitude oscillatory and step strain measurements. The onset stress for stress-stiffening (σ^*) is also higher for $Plec^{+/+}$ cells (22 ±5 Pa) compared with $Plec^{-/-}$ cells (13 ±5 Pa), consistent with their higher modulus (Fig. 5 *E*, right). The corresponding onset strains for strain-stiffening (ϵ^*) are therefore only slightly different between $Plec^{+/+}$ cells (0.063 ±0.008) versus $Plec^{-/-}$ cells (0.042 ± 0.004) (Fig. 5 *E*, middle).

Repeated compression softens wild-type cells more than plectin knockout cells

In many organs, such as the heart, skin, and lungs, cells are subject to repeated compression. To test whether plectin knockout changes the mechanical response of fibroblasts upon repeated loading, we applied five subsequent linear compression-decompression strain ramps with an amplitude of 5 μm and strain rate of 1 μm/s (Fig. 6 *A*). Fig. 6 *B* shows a typical example of the strain (yellow) and stress (purple) for a $Plec^{+/+}$ cell. In each subsequent cycle, we see that the stresses decrease for every given strain. To rule out the possibility that this softening was due to cell flattening, we performed widefield fluorescence imaging to monitor for possible changes in cell diameter or other morphological features. We found that the cell diameter returned to its original value after each cycle, indicating that no gross shape

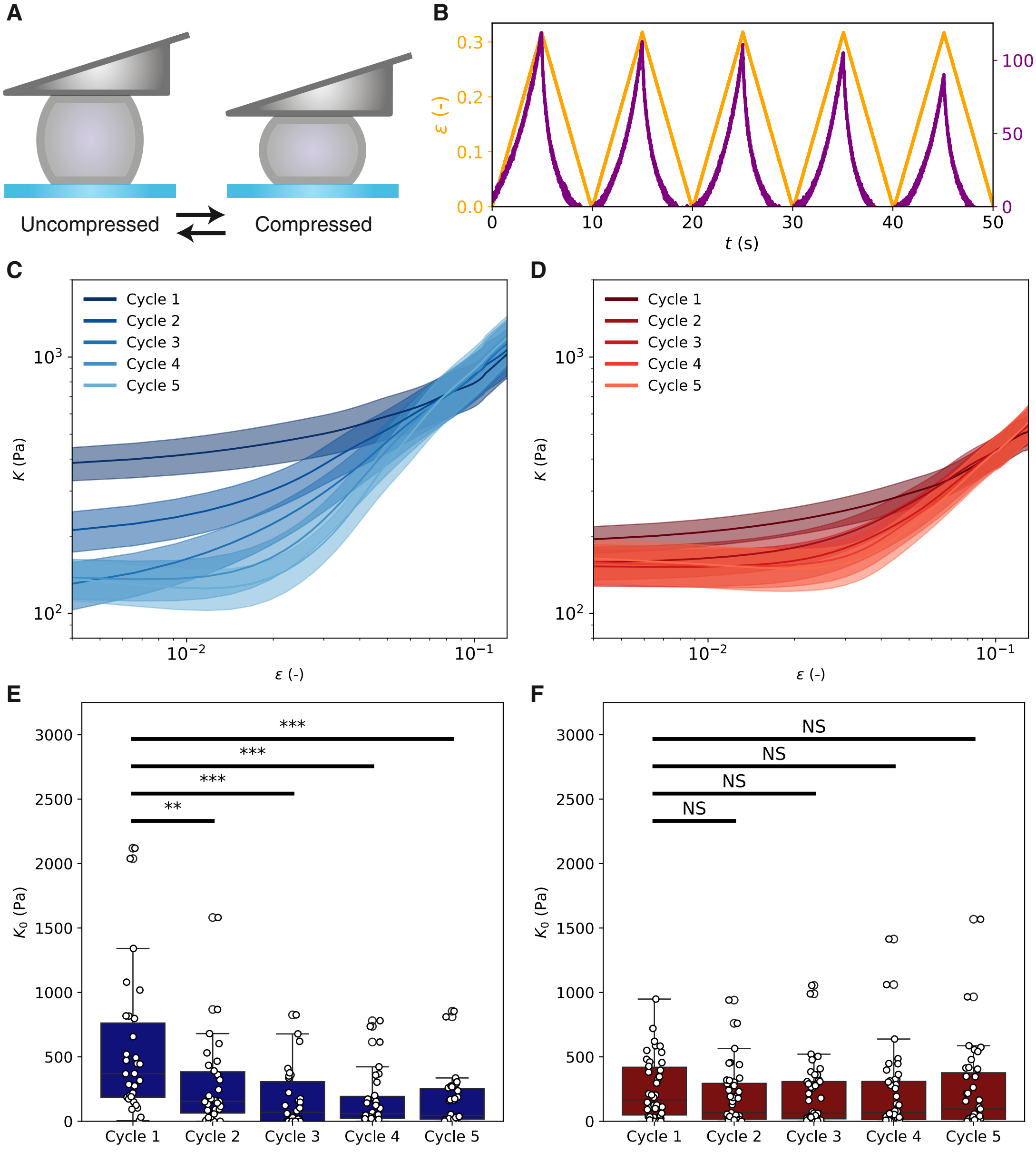

FIGURE 6 Mechanical response of $Plec^{+/+}$ and $Plec^{-/-}$ cells to repeated compressive loading-unloading cycles. (*A*) Schematic showing toggling between an uncompressed state (zero strain) and compressed state (strain ~0.4) by gradual lowering and raising of the cantilever. (*B*) Example measurement on a $Plec^{+/+}$ cell showing the applied strain (*yellow, left y-axis*) and measured stress (*purple, right y-axis*). For each subsequent cycle, the stress decreases. (*C*) Averaged dependence of the differential modulus K on applied strain for $Plec^{+/+}$ cells ($N = 30$). We observe a large reduction in K at small strain between the first and fifth cycle of compression, whereas the curves converge at high strain. (*D*) Average K/ϵ curves for $Plec^{-/-}$ cells ($N = 42$), showing little change in the modulus between cycles. Error bands in (*C*) and (*D*) represent the standard error. (*E*) Linear modulus K_0 as a function of cycle number for $Plec^{+/+}$ cells obtained from fitting data for individual cells (shown in Fig. S9). (*F*) K_0 as a function of cycle number for $Plec^{-/-}$ cells. Note that the $Plec^{+/+}$ cells show significant softening upon repeated loading (** denotes $p < 0.01$, *** denotes $p < 0.001$), whereas the $Plec^{-/-}$ cells do not soften appreciably (differences N.S.).

changes occurred with repeated compression (Fig. S10). The cell contours remained smooth as long as the strain remained below 0.5, beyond which point cells often started to bleb as a sign of damage.

To quantify the degree of softening upon repeated loading-unloading cycles, we calculated the differential stiffness K for each cycle of deformation, following the same procedure as for the single-cycle compressive ramps. For $Plec^{+/+}$ cells, we found a substantial reduction in cell stiffness in the linear regime between the first and second cycle, with a further gradual reduction as cells were subjected to additional cycles (Fig. 6 *C*). At high stress, the stress-strain curves converged. For $Plec^{-/-}$ cells, we found qualitatively similar behavior, but the reduction of the

stiffness in the linear regime between the first and subsequent cycles was comparatively minor (Fig. 6 *D*). To better visualize this, we tracked the value of the linear modulus K_0 as a function of cycle number. For $Plec^{+/+}$ cells, K_0 was 400 $\pm$70 Pa in the first cycle and only 120 $\pm$30 Pa in the fifth cycle (Fig. 6 *E*). By contrast, $Plec^{-/-}$ cells displayed a comparable linear modulus throughout the cycles, with average K_0 values of 180 $\pm$30 Pa for the first cycle and 160 $\pm$40 Pa for the fifth cycle (Fig. 6 *F*). Interestingly, these stiffness values are comparable to the stiffness of $Plec^{+/+}$ cells after repeated compression, strongly suggesting that the plectin cross-linkers that contribute to the modulus initially are disrupted by large compressive deformations.

DISCUSSION

The aim of this study was to determine the contribution of plectin to the viscoelastic response of fibroblasts at small and large deformations using a custom single-cell rheometer. We first assessed the linear viscoelastic response using two complementary assays: small-amplitude oscillatory compressions and stress relaxation measurements. Both assays independently confirmed that fibroblasts behave as viscoelastic solids, displaying an elastic Young's modulus, $E'(f)$, that is larger than the loss modulus, $E''(f)$, with weak dependencies of the moduli on frequency. We next applied large uniaxial strains to investigate the nonlinear elasticity of the cells. Under these conditions, the cells showed pronounced strain-stiffening, with the differential elastic modulus rising sharply beyond $\sim$4% compressive strain. Both the small and large amplitude experiments showed that plectin knockout makes the mouse embryonic fibroblasts about twofold stiffer. This finding is in line with previous measurements on mouse skin fibroblasts (35) and mouse myoblasts (37). These studies used localized measurement methods in contrast to our whole-cell compression experiments but also found $Plec^{-/-}$ cells to be softer than $Plec^{+/+}$ cells (see Table 1). However, other studies reported a modest 10% increase in stiffness upon plectin knockout in mouse keratinocytes (37) and no significant change in vulvar carcinoma cells (38). These contradictory findings could stem from variations in cell lines and cell types used and/or from the different measurement techniques employed.

A more detailed interpretation of our data aided by available theoretical models showed intriguing effects of plectin on the time- and amplitude-dependent mechanical response of fibroblasts. First, the stress relaxation measurements revealed that plectin reduced the poroelastic timescale that characterizes stress relaxation via intracellular fluid flow at short times (<1 s) after a rapid compression (62). By also measuring the cytosolic viscosity by FRAP measurements on a small cytoplasmic dye, we found that this effect is caused by a larger pore size of the solid phase of the cytoplasm. The solid phase comprises the cytoskeleton, nucleus, and other organelles. Confocal imaging and flow cytometry showed that the wild-type and plectin knockout cells had similar sizes and also similarly sized nuclei. However, the organization of the vimentin network was drastically different, with a dense meshwork in wild-type cells versus a sparse network of bundles in plectin knockout cells. This bundling is expected to increase the pore size of the cytoskeletal matrix, which should indeed enhance the rate of poroelastic relaxation. Our data therefore suggest that plectin impacts poroelastic relaxation by regulating the organization of the vimentin cytoskeleton. This could be a consequence of its cross-linking role, both as a vimentin-vimentin and vimentin-actin cross-linker. In addition, anchoring of the vimentin network to focal adhesions and the nucleus by plectin could also help prevent vimentin filaments from collapsing into bundles.

A second finding from the stress relaxation experiments was that viscoelastic stress relaxation at times beyond the poroelastic regime obeys a fractional rheology model. According to this model, the cells display power law rheology for frequencies between 0.1 Hz and 10 Hz, characterized by a weak power law dependence of $E'(f)$ on f. Power law rheology signifies a broad distribution of characteristic timescales and is typical of mammalian cells (53). On longer timescales, the model includes an additional stress relaxation mechanism characterized by an exponential dependence on time. The same fractional rheology model could also self-consistently capture the frequency dependence of both the elastic and viscous moduli measured through oscillatory measurements. This consistency suggests that this model, though phenomenological, provides an adequate description of the main mechanisms contributing to the cell's viscoelastic response. By fitting the data, we could compare the corresponding material parameters between $Plec^{+/+}$ and $Plec^{-/-}$ cells.

We found that removal of plectin altered the elastic component of the complex modulus, marked by a reduction in the elastic parameter k. It did not significantly change the power law scaling exponent β describing the frequency dependence at intermediate times. This result indicates that plectin's cross-linking function predominantly reinforces the elastic integrity of the cytoskeletal network, whereas the fundamental mechanisms governing energy dissipation at intermediate timescales remain intact. This is consistent with previous studies on reconstituted actin networks with cross-linkers (78), which demonstrated that increasing the cross-linker density markedly enhances the elastic stiffness, whereas the frequency-dependent power law scaling of the viscoelastic response remains largely unchanged. However, at longer timescales ($>\sim$10 s), we found faster stress relaxation for $Plec^{-/-}$ cells than for $Plec^{+/+}$ cells. At these long times, we expect stress relaxation to be tuned by the active turnover of the cytoskeletal filaments and/or rearrangements of the cytoskeleton via cross-linker exchange or myosin contractility (73).

TABLE 1 Summary of previously reported changes in the stiffness of single cells at small deformations upon plectin knockout for different cell model systems and with different measurement techniques

Reference	Cell model	Measurement technique	Change in cell stiffness
Na et al. (35)	Mouse skin fibroblasts[a,c]	Magnetic twisting cytometry	$Plec^{-/-}$ cells ∼30%–60% softer
Bonakdar et al.(37)	Mouse myoblasts[a,d]	Magnetic tweezers microrheology	$Plec^{-/-}$ cells ∼50% softer
Bonakdar et al. (37)	Mouse keratinocytes[a,d]	Magnetic tweezers microrheology	$Plec^{-/-}$ cells ∼10% stiffer
Moch et al. (38)	Vulvar carcinoma cells[b,d]	Atomic force microscopy	No change

[a]Knockout model system.
[b]Knockdown model system.
[c]Primary cell system.
[d]Cell model system.

Interestingly, FRAP experiments on actin-GFP transfected cells demonstrated that actin turnover was faster in plectin knockout cells and coincided with the timescale of stress relaxation in step strain experiments. Taken together, this suggests that plectin changes viscoelastic relaxation mainly by impacting actin turnover. This mirrors findings in cells (79–81) and in vitro (82) where dynamic cross-linkers (e.g., α-actinin, filamin) slow actin network relaxation by stabilizing filament overlaps and reducing filament turnover, underscoring that plectin's cross-linking-mediated control of actin dynamics is a key determinant of cytoplasmic viscoelasticity. This is likely via cross-linking of actin to vimentin but potentially also to actin, since plectin is dimeric (36). Intriguingly, a similar increase in actin turnover has also been observed in vimentin knockout cells, where the absence of vimentin led to enhanced phosphorylation of GEF-H1 at Ser886, thereby elevating RhoA activity and promoting stress fiber assembly, which in turn accelerates actin dynamics (83). However, we note that the environmental conditions in those experiments, where cells were adherent to flat substrates, are different from our experiments, where the cells were confined between nonadhesive plates. Consistent with these nonadherent conditions, confocal imaging did not reveal clear signs of stress fibers and only showed a fine actin meshwork.

In this study, we deliberately examined nonadherent fibroblasts in suspension to eliminate substrate adhesion and associated contractile prestress. This reductionist strategy isolates the intrinsic mechanical contributions of the cytoskeletal networks—particularly actin and vimentin (42)—without the confounding effects of focal adhesion signaling, stress fiber formation, and cortical tension. Although adhesion and contractility are physiologically important, they induce dynamic remodeling and fluidization that can obscure the baseline mechanical behavior of the cell interior (84). Our approach therefore provides a well-defined experimental limit to benchmark cytoskeletal mechanics. Moving forward, it will be important to understand how adhesion-mediated cues modulate these intrinsic properties using 2D as well as 3D conditions. This work also highlights the need for new theoretical models that capture the mechanical interplay between the actin cortex, cytoplasmic intermediate filament networks, and the enclosed nucleus. The measured mechanical response to uniaxial compression will capture a composite mechanical response of the cell interior, the cortical actin shell, and the plasma membrane. How to appropriately model a cell as a composite system with an active cortex, viscoelastic interior, and deformable nuclear compartment remains an open and pressing question.

Finally, our strain ramp experiments confirmed that plectin-deficient cells were softer than wild-type cells at small deformations. However, at larger strains, both cell types showed stress-stiffening, characterized by a marked increase of the differential stiffness beyond a critical deformation. Notably, at large deformations, not previously accessed in other studies of the mechanical impact of plectin, plectin knockout cells reached a similar stiffness as wild-type cells. The modulus-strain curves converged at high stress and showed a similar scaling with stress, suggesting that the mechanism of nonlinearity is the same. Actin and vimentin filaments are both semiflexible polymers. Theoretical models predict such semiflexible polymer networks to strain-stiffen due to the entropic cost of pulling out thermal bending fluctuations (85,86). For uniform dense networks, models predict an affine response with modulus increasing with stress as $\sigma^{3/2}$, consistent with experiments on reconstituted actin and intermediate filament networks (87,88). In our experiments the stiffening response tended to a weaker scaling, with an exponent between 0.5 and 1. This could potentially signify a nonaffine response. The comparable stress-stiffening response of wild-type and plectin knockout cells revealed by the collapse of our data onto a master curve suggests that the processes underlying nonlinear elasticity remain largely unaffected by plectin's absence. We speculate that strain-stiffening in $Plec^{-/-}$ cells versus $Plec^{+/+}$ cells is nearly identical because, at high strains, the mechanical response primarily depends on polymer content and the stretching of the network, rather than on specific cross-linkers. Cross-linker proteins mainly influence linear elasticity and the onset strain for nonlinearity by changing the amount of excess filament length between filament junctions—as established in reconstituted cytoskeletal networks (87,88). Strain stiffening requires the presence of cross-linkers for enabling nonlinear stiffening and preventing flow, but in the cell, this role need not be fulfilled

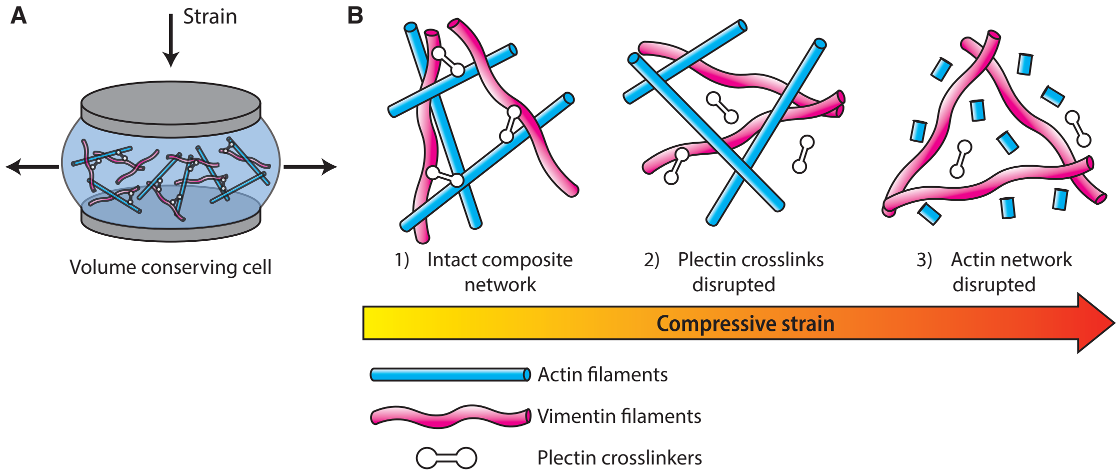

FIGURE 7 Proposed conceptual model for the impact of plectin on the strain-dependent mechanical response of single cells under compression. (*A*) Upon uniaxial compression, due to its incompressible nature, the cell bulges at the edges, leading to stretching of the cytoskeleton. (*B*) Starting with an intact composite actin-vimentin network cross-linked via plectin (*left*), stretching of the cytoskeleton first leads to the disruption of plectin cross-linking (*middle*). With further increasing compressive strain, the actin network is disrupted, leaving only the more mechanically resilient vimentin network intact (*right*).

exclusively by an actin-vimentin cross-linker like plectin, as alternative actin cross-linkers are abundant and sufficient at high strain. Thus, even without plectin, these other cross-link proteins can sustain the nonlinear response, although plectin itself notably impacts mechanical behavior at lower stress levels.

Strain ramps with repeated loading-unloading cycles revealed another interesting effect of plectin at large deformations. Wild-type cells that had not yet been compressed had a higher linear modulus than plectin knockout cells, but after one or more additional cycles, the wild-type cells became about as soft as plectin knockout cells. By contrast, the plectin knockout cells did not soften significantly during repeated loading. This finding suggests that plectin cross-linkers initially contribute to cell stiffness in wild-type cells but are disrupted by large compressive deformations. We speculate that this could suggest a model where plectin contributes an important mechanism of mechanical protection of the cytoskeleton (Fig. 7). Upon uniaxial compression, due to its incompressible nature, the cell bulges at the edges, leading to stretching of the cytoskeleton (Fig. 7 *A*). In an unperturbed cell, actin and vimentin networks interpenetrate and are cross-linked by plectin (Fig. 7 *B*). With increasing deformations, stretching of the cytoskeleton first leads to the disruption of plectin cross-linking. We are not aware of any measurements of the plectin binding and unbinding rates to the cytoskeleton in cells. However, measurements on a biochemically reconstituted system showed that the PRD domains of plectin bind quite strongly to vimentin filaments, with a dissociation constant, k_d, of $0.5 - 0.9 \times 10^{-7}$ M (89), whereas the ABD binds more weakly to actin filaments ($k_d = 3.2 \times 10^{-7}$ M) (90). Even if plectin could exhibit catch bond behavior like various actin-binding proteins (13), at sufficiently large mechanical load, we expect plectin bonds to dissociate. With further increasing compressive strain, most likely the actin network is disrupted first. The more mechanically resilient vimentin network can likely remain intact, as indicated by measurements on reconstituted networks and on cells showing that intermediate filaments can sustain large ($>200\%$) strains (91), whereas actin filaments and/or actin-cross-links are expected to rupture first under compressive loads (92). In situ imaging of the actin and vimentin cytoskeletons under compression could allow us to directly test this prediction.

Our results suggest that plectin contributes primarily to the elastic reinforcement of the cytoskeletal network at small deformations, while having minimal effect on the power law rheology and stress-stiffening of fibroblasts. Cross-linking via plectin, mainly thought to be between actin and vimentin (19), makes a significant contribution to the rheology of fibroblasts. However, plectin's multifaceted roles complicate the interpretation of its mechanical contributions. In addition to cross-linking actin and intermediate filaments, plectin is known to interact with the linker of nucleoskeleton and cytoskeleton complex (93,94)—thereby linking the nucleus to the cytoskeleton—and to localize at focal adhesions where it contributes to adhesion dynamics and force transmission (35,66). Consequently, the observed reduction in stiffness upon plectin deficiency may result not only from the loss of its cross-linking activity but also from disrupted connections at the nuclear envelope and altered focal adhesion mechanics. Future studies employing domain-specific manipulations will be essential to disentangle these distinct functions and to clarify the relative contributions of plectin's various binding interactions to cellular mechanics.

CONCLUSION

In this study, we have shown that plectin knockout ($Plec^{-/-}$) fibroblasts are twice as soft as their wild-type ($Plec^{+/+}$) counterparts at small and large deformations, but their strain-stiffening response to compression is similar. Applying a step strain led to poroelastic stress relaxation at short times and a nonexponential response at long times with faster stress relaxation for $Plec^{-/-}$ cells compared with $Plec^{+/+}$ cells. We hypothesize that this difference is due to a threefold difference in the kinetics of F-actin network turnover in the absence of plectin-mediated cross-linking to vimentin. When cells were subjected to repeated compression cycles, wild-type fibroblasts progressively softened to stiffness levels comparable to those of plectin knockout cells, indicating that plectin provides a first line of defense against large compressive loads. Confocal fluorescence images of the cells revealed an altered vimentin cytoskeletal network for $Plec^{-/-}$ cells, where vimentin intermediate filaments formed bundles, rather than the finer meshwork displayed in $Plec^{+/+}$ cells.

In conclusion, our findings reveal that plectin cross-linking significantly affects the cytoskeletal organization and viscoelastic properties of fibroblasts, at least partially through cross-linking of the actin and vimentin cytoskeletal networks. These findings provide a starting point toward understanding the role of mechanical integration of the different cytoskeletal networks in the cell interior and emphasize that these networks cannot be regarded as separate entities. Future work using engineered cross-linkers to selectively restore specific binding functions of plectin (19) could help disentangle its mechanical roles and reveal how distinct cross-linking interactions contribute to cytoskeletal mechanics.

ACKNOWLEDGMENTS

We thank M. Mavrakis (Institut Fresnel) for the vimentin-GFP plasmid used for live cell imaging experiments, C. Silva Martins (Institut Fresnel) for help with electroporation, and G. Charras (University College London) for the actin-GFP plasmid used for FRAP measurements. We thank F. Ramirez Gomez (TU Delft) for help with flow cytometry measurements and C. Sharma (TU Delft) for helping with FRAP experiments. We thank A. Bonfanti (Politecnico di Milano) for valuable help and advice regarding the RHEOS Julia package. G.H.K. gratefully acknowledges funding from the NWO Talent Programme, which is financed by the Dutch Research Council (project number VI.C.182.004). L.W. was funded by Austrian Science Fund (FWF) grants P31541-B27 (grant-DOI 10.55776/P31541) and I6049-B (grant-DOI 10.55776/I6049). F.C.M. was supported in part by the National Science Foundation Division of Materials Research (grant no. DMR-2224030) and the Center for Theoretical Biological Physics (grant no. PHY-2019745).

AUTHOR CONTRIBUTIONS

J.P.C., M.G.L, F.C.M., and G.H.K. designed the research. J.P.C., M.G.L., N. v.V., and L.W. performed research. J.P.C., M.G.L., F.C.M., and G.H.K wrote the manuscript. L.W. and G.W. provided the cellular model systems and contributed to data interpretation. All authors edited the manuscript.

DECLARATION OF INTERESTS

The authors declare no competing interests.

SUPPORTING MATERIAL

Supporting material can be found online at https://doi.org/10.1016/j.bpj.2025.09.001.

REFERENCES

1. Urbanska, M., and J. Guck. 2024. Single-Cell Mechanics: Structural Determinants and Functional Relevance. *Annu. Rev. Biophys.* 53:367–395.
2. Conboy, J. P., I. Istúriz Petitjean, …, G. H. Koenderink. 2024. How cytoskeletal crosstalk makes cells move: Bridging cell-free and cell studies. *Biophys. Rev.* 5:021307.
3. Nöding, B., and S. Köster. 2012. Intermediate filaments in small configuration spaces. *Phys. Rev. Lett.* 108:1–4.
4. Bridges, A. A., H. Zhang, …, A. S. Gladfelter. 2014. Septin assemblies form by diffusion-driven annealing on membranes. *Proc. Natl. Acad. Sci. USA.* 111:2146–2151.
5. Gittes, F., B. Mickey, …, J. Howard. 1993. Flexural rigidity of microtubules and actin filaments measured from thermal fluctuations in shape. *J. Cell Biol.* 120:923–934.
6. Lappalainen, P., T. Kotila, …, G. Romet-Lemonne. 2022. Biochemical and mechanical regulation of actin dynamics. *Nat. Rev. Mol. Cell Biol.* 23:836–852.
7. Akhmanova, A., and L. C. Kapitein. 2022. Mechanisms of microtubule organization in differentiated animal cells. *Nat. Rev. Mol. Cell Biol.* 23:541–558.
8. Coelho-Rato, L. S., S. Parvanian, …, J. E. Eriksson. 2024. Intermediate filaments at a glance. *J. Cell Sci.* 137:4345–4347.
9. Schampera, J. N., and C. Schwan. 2024. Septin dynamics and organization in mammalian cells. *Curr. Opin. Cell Biol.* 91:102442.
10. Lieleg, O., M. M. A. E. Claessens, and A. R. Bausch. 2010. Structure and dynamics of cross-linked actin networks. *Soft Matter.* 6:218–225.
11. Müller, K. W., R. F. Bruinsma, …, A. J. Levine. 2014. Rheology of semiflexible bundle networks with transient linkers. *Phys. Rev. Lett.* 112:1–5.
12. Broedersz, C. P., M. Depken, …, F. C. MacKintosh. 2010. Cross-link-governed dynamics of biopolymer networks. *Phys. Rev. Lett.* 105:238101.
13. Mulla, Y., M. J. Avellaneda, …, G. H. Koenderink. 2022. Weak catch bonds make strong networks. *Nat. Mater.* 21:1019–1023.
14. Suozzi, K. C., X. Wu, and E. Fuchs. 2012. Spectraplakins: Master orchestrators of cytoskeletal dynamics. *J. Cell Biol.* 197:465–475.
15. Zhang, J., J. Yue, and X. Wu. 2017. Spectraplakin family proteins - Cytoskeletal crosslinkers with versatile roles. *J. Cell Sci.* 130:2447–2457.
16. Pytela, R., and G. Wiche. 1980. High molecular weight polypeptides (270,000-340,000) from cultured cells are related to hog brain microtubule-associated proteins but copurify with intermediate filaments. *Proc. Natl. Acad. Sci. USA.* 77:4808–4812.
17. Foisner, R., F. E. Leichtfried, …, G. Wiche. 1988. Cytoskeleton-associated plectin: in situ localization, in vitro reconstitution, and binding to immobilized intermediate filament proteins. *J. Cell Biol.* 106:723–733.
18. Liu, C. G., C. Maercker, …, G. Wiche. 1996. Human plectin: Organization of the gene, sequence analysis, and chromosome localization (8q24). *Proc. Natl. Acad. Sci. USA.* 93:4278–4283.
19. Wiche, G. 2021. Plectin-Mediated Intermediate Filament Functions: Why Isoforms Matter. *Cells.* 10:2154.

20. Svitkina, T. M., A. B. Verkhovsky, and G. B. Borisy. 1998. Plectin sidearms mediate interactions of intermediate filaments with microtubules and other components of the cytoskeleton. *Biol. Bull.* 194:409–410.
21. Ruiz, W. G., D. R. Clayton, …, G. Apodaca. 2024. The rat bladder umbrella cell keratin network: Organization, dependence on the plectin cytolinker, and responses to bladder filling. *Mol. Biol. Cell.* 35:ar139.
22. Osmanagic-Myers, S., S. Rus, …, G. Wiche. 2015. Plectin reinforces vascular integrity by mediating crosstalk between the vimentin and the actin networks. *J. Cell Sci.* 128:4138–4150.
23. Prechova, M., Z. Adamova, …, M. Gregor. 2022. Plectin-mediated cytoskeletal crosstalk controls cell tension and cohesion in epithelial sheets. *J. Cell Biol.* 221:e202105146.
24. Jirouskova, M., K. Nepomucka, …, M. Gregor. 2018. Plectin controls biliary tree architecture and stability in cholestasis. *J. Hepatol.* 68:1006–1017.
25. Winter, L., and G. Wiche. 2013. The many faces of plectin and plectinopathies: pathology and mechanisms. *Acta Neuropathol.* 125:77–93.
26. Kiritsi, D., L. Tsakiris, and F. Schauer. 2021. Plectin in Skin Fragility Disorders. *Cells.* 10:2738.
27. Zrelski, M. M., M. Kustermann, and L. Winter. 2021. Muscle-Related Plectinopathies. *Cells.* 10:2480.
28. Zrelski, M. M., S. Hösele, …, L. Winter. 2024. Plectin Deficiency in Fibroblasts Deranges Intermediate Filament and Organelle Morphology, Migration, and Adhesion. *J. Invest. Dermatol.* 144:547–562.e9.
29. Osmanagic-Myers, S., M. Gregor, …, G. Wiche. 2006. Plectin-controlled keratin cytoarchitecture affects MAP kinases involved in cellular stress response and migration. *J. Cell Biol.* 174:557–568.
30. Serres, M. P., M. Samwer, …, E. K. Paluch. 2020. F-Actin Interactome Reveals Vimentin as a Key Regulator of Actin Organization and Cell Mechanics in Mitosis. *Dev. Cell.* 52:210–222.e7.
31. Duarte, S., Á. Viedma-Poyatos, …, D. Pérez-Sala. 2019. Vimentin filaments interact with the actin cortex in mitosis allowing normal cell division. *Nat. Commun.* 10:4200.
32. Sutoh Yoneyama, M., S. Hatakeyama, …, S. Tsuboi. 2014. Vimentin intermediate filament and plectin provide a scaffold for invadopodia, facilitating cancer cell invasion and extravasation for metastasis. *Eur. J. Cell Biol.* 93:157–169.
33. Jiu, Y., J. Lehtimäki, …, P. Lappalainen. 2015. Bidirectional Interplay between Vimentin Intermediate Filaments and Contractile Actin Stress Fibers. *Cell Rep.* 11:1511–1518.
34. Marks, P. C., B. R. Hewitt, …, R. J. Petrie. 2022. Plectin linkages are mechanosensitive and required for the nuclear piston mechanism of three-dimensional cell migration. *Mol. Biol. Cell.* 33:ar104.
35. Na, S., F. Chowdhury, …, N. Wang. 2009. Plectin contributes to mechanical properties of living cells. *Am. J. Physiol. Cell Physiol.* 296:868–877.
36. Petitjean, I. I., Q. D. Tran, …, G. H. Koenderink. 2024. Reconstitution of cytolinker-mediated crosstalk between actin and vimentin. *Eur. J. Cell Biol.* 103:151403.
37. Bonakdar, N., A. Schilling, …, W. H. Goldmann. 2015. Determining the mechanical properties of plectin in mouse myoblasts and keratinocytes. *Exp. Cell Res.* 331:331–337.
38. Moch, M., R. Windoffer, …, R. E. Leube. 2016. Effects of plectin depletion on keratin network dynamics and organization. *PLoS One.* 11:e0149106.
39. Wu, P.-H., D. R.-B. Aroush, …, D. Wirtz. 2018. A comparison of methods to assess cell mechanical properties. *Nat. Methods.* 15:491–498.
40. Fabry, B., G. N. Maksym, …, J. J. Fredberg. 2003. Timescale and other invariants of integrative mechanical behavior in living cells. *Phys. Rev. E - Stat. Nonlinear Soft Matter Phys.* 68:041914.
41. Guo, M., A. J. Ehrlicher, …, D. A. Weitz. 2013. The role of vimentin intermediate filaments in cortical and cytoplasmic mechanics. *Biophys. J.* 105:1562–1568.
42. Conboy, J. P., M. G. Lettinga, …, G. H. Koenderink. 2025. Actin and vimentin jointly control cell viscoelasticity and compression stiffening. Preprint at bioRxiv. https://doi.org/10.1101/2025.01.01.630993.
43. Andrä, K., H. Lassmann, …, G. Wiche. 1997. Targeted inactivation of plectin reveals essential function in maintaining the integrity of skin, muscle, and heart cytoarchitecture. *Gene Dev.* 11:3143–3156.
44. Andrä, K., I. Kornacker, …, G. Wiche. 2003. Plectin-isoform-specific rescue of hemidesmosomal defects in plectin keratinocytes. *J. Invest. Dermatol.* 120:189–197.
45. Burgstaller, G., M. Gregor, …, G. Wiche. 2010. Keeping the vimentin network under control: cell-matrix adhesion-associated plectin 1f affects cell shape and polarity of fibroblasts. *Mol. Biol. Cell.* 21:3362–3375.
46. Van Hoorn, H., N. A. Kurniawan, …, D. Iannuzzi. 2016. Local dynamic mechanical analysis for heterogeneous soft matter using ferrule-top indentation. *Soft Matter.* 12:3066–3073.
47. Llewellyn, M. E., R. P. J. Barretto, …, M. J. Schnitzer. 2008. Minimally invasive high-speed imaging of sarcomere contractile dynamics in mice and humans. *Nature.* 454:784–788.
48. Shikano, K., K. Chiba, and S. Miyata. 2015. Response of Human Skin Fibroblasts to Mechanical Stretch in Wound Healing Process Analyzed Using a Three-Dimensional Culture Model. *Advanced Biomedical Engineering.* 4:170–178.
49. Seliktar, D., R. A. Black, …, R. M. Nerem. 2000. Dynamic Mechanical Conditioning of Collagen-Gel Blood Vessel Constructs Induces Remodeling In Vitro. *Ann. Biomed. Eng.* 28:351–362.
50. Van Der Walt, S., J. L. Schönberger, …, T. Yu. 2014. Scikit-image: Image processing in python. *PeerJ.* 2014:1–18.
51. Virtanen, P., R. Gommers, …, Y. Vázquez-Baeza. 2020. SciPy 1.0: fundamental algorithms for scientific computing in Python. *Nat. Methods.* 17:261–272.
52. Kaplan, J., A. Bonfanti, and A. Kabla. 2019. RHEOS.jl – A Julia Package for Rheology Data Analysis. *J. Open Source Softw.* 4:1700.
53. Fabry, B., G. N. Maksym, …, J. J. Fredberg. 2001. Scaling the microrheology of living cells. *Phys. Rev. Lett.* 87:148102.
54. Hurst, S., B. E. Vos, …, T. Betz. 2021. Intracellular softening and increased viscoelastic fluidity during division. *Nat. Phys.* 17:1270–1276.
55. Bonfanti, A., J. L. Kaplan, …, A. Kabla. 2020. Fractional viscoelastic models for power-law materials. *Soft Matter.* 16:6002–6020.
56. Bonfanti, A., J. Fouchard, …, A. Kabla. 2020. A unified rheological model for cells and cellularised materials. *R. Soc. Open Sci.* 7:190920.
57. Chan, E. P., B. Deeyaa, …, C. M. Stafford. 2012. Poroelastic relaxation of polymer-loaded hydrogels. *Soft Matter.* 8:8234–8240.
58. Harris, C. R., K. J. Millman, …, T. E. Oliphant. 2020. Array programming with NumPy. *Nature.* 585:357–362.
59. Leduc, C., and S. Etienne-Manneville. 2017. Regulation of microtubule-associated motors drives intermediate filament network polarization. *J. Cell Biol.* 216:1689–1703.
60. Schindelin, J., I. Arganda-Carreras, …, A. Cardona. 2012. Fiji: An open-source platform for biological-image analysis. *Nat. Methods.* 9:676–682.
61. Sharma, V. P., B. Tang, …, M. H. Oktay. 2021. Live tumor imaging shows macrophage induction and TMEM-mediated enrichment of cancer stem cells during metastatic dissemination. *Nat. Commun.* 12:7300–7324.
62. Moeendarbary, E., L. Valon, …, G. T. Charras. 2013. The cytoplasm of living cells behaves as a poroelastic material. *Nat. Mater.* 12:253–261.
63. Kang, M., C. A. Day, …, E. DiBenedetto. 2009. A generalization of theory for two-dimensional fluorescence recovery after photobleaching applicable to confocal laser scanning microscopes. *Biophys. J.* 97:1501–1511.
64. Vázquez-Meza, H., M. M. Vilchis-Landeros, …, D. Matuz-Mares. 2023. Cellular Compartmentalization, Glutathione Transport and Its Relevance in Some Pathologies. *Antioxidants.* 12:834.

65. Lowry, O. H., N. J. Rosebrough, …, R. J. Randall. 1951. Protein measurement with the Folin phenol reagent. *J. Biol. Chem.* 193:265–275.

66. Burgstaller, G., M. Gregor, …, G. Wiche. 2010. Keeping the Vimentin Network under Control: Cell-Matrix Adhesion-associated Plectin 1f Affects Cell Shape and Polarity of Fibroblasts. *Mol. Biol. Cell.* 21:3362–3375.

67. Patteson, A. E., A. Vahabikashi, …, P. A. Janmey. 2019. Vimentin protects cells against nuclear rupture and DNA damage during migration. *J. Cell Biol.* 218:4079–4092.

68. Kollmannsberger, P., and B. Fabry. 2011. Linear and Nonlinear Rheology of Living Cells. *Annu. Rev. Mater. Res.* 41:75–97.

69. Kondo, A., and J. G. Susset. 1973. Physical properties of the urinary detrusor muscle: A mechanical model based upon the analysis of stress relaxation curve. *J. Biomech.* 6:141–151.

70. Oftadeh, R., B. K. Connizzo, …, A. J. Grodzinsky. 2018. Biological connective tissues exhibit viscoelastic and poroelastic behavior at different frequency regimes: Application to tendon and skin biophysics. *Acta Biomater.* 70:249–259.

71. Mason, T. G., and D. A. Weitz. 1995. Optical Measurements of Frequency-Dependent Linear Viscoelastic Moduli of Complex Fluids. *Phys. Rev. Lett.* 74:1250–1253.

72. Cantor, C. R., and P. R. Schimmel. 1980. *In* Biophysical Chemistry. Part. 1: The Conformation of Biological Macromolecules, 1st edition W. H. Freeman.

73. Khalilgharibi, N., J. Fouchard, …, G. Charras. 2019. Stress relaxation in epithelial monolayers is controlled by the actomyosin cortex. *Nat. Phys.* 15:839–847.

74. Watanabe, N., and T. J. Mitchison. 2002. Single-molecule speckle analysis of actin filament turnover in lamellipodia. *Science.* 295:1083–1086.

75. Fritzsche, M., A. Lewalle, …, G. Charras. 2013. Analysis of turnover dynamics of the submembranous actin cortex. *Mol. Biol. Cell.* 24:757–767.

76. Robert, A., M. J. Rossow, …, V. I. Gelfand. 2015. Vimentin filament precursors exchange subunits in an ATP-dependent manner. *Proc. Natl. Acad. Sci. USA.* 112:E3505–E3514.

77. Gandikota, M. C., K. Pogoda, …, J. M. Schwarz. 2020. Loops: Versus lines and the compression stiffening of cells. *Soft Matter.* 16:4389–4406.

78. Gardel, M. L., F. Nakamura, …, D. A. Weitz. 2006. Prestressed F-actin networks cross-linked by hinged filamins replicate mechanical properties of cells. *Proc. Natl. Acad. Sci. USA.* 103:1762–1767.

79. Li, A., J. C. Dawson, …, L. M. Machesky. 2010. The Actin-Bundling Protein Fascin Stabilizes Actin in Invadopodia and Potentiates Protrusive Invasion. *Curr. Biol.* 20:339–345.

80. Chaubet, L., A. R. Chaudhary, …, A. G. Hendricks. 2020. Dynamic actin cross-linking governs the cytoplasm's transition to fluid-like behavior. *Mol. Biol. Cell.* 31:1744–1752.

81. Mukhina, S., Y.-L. Wang, and M. Murata-Hori. 2007. α-Actinin Is Required for Tightly Regulated Remodeling of the Actin Cortical Network during Cytokinesis. *Dev. Cell.* 13:554–565.

82. Lieleg, O., J. Kayser, …, A. R. Bausch. 2011. Slow dynamics and internal stress relaxation in bundled cytoskeletal networks. *Nat. Mater.* 10:236–242.

83. Jiu, Y., J. Peränen, …, P. Lappalainen. 2017. Vimentin intermediate filaments control actin stress fiber assembly through GEF-H1 and RhoA. *J. Cell Sci.* 130:892–902.

84. Nietmann, P., J. E. F. Bodenschatz, …, A. Janshoff. 2022. Epithelial cells fluidize upon adhesion but display mechanical homeostasis in the adherent state. *Biophys. J.* 121:361–373.

85. Broedersz, C. P., and F. C. Mackintosh. 2014. Modeling semiflexible polymer networks. *Rev. Mod. Phys.* 86:995–1036.

86. MacKintosh, F. C., J. Käs, and P. A. Janmey. 1995. Elasticity of semiflexible biopolymer networks. *Phys. Rev. Lett.* 75:4425–4428.

87. Gardel, M. L., J. H. Shin, …, D. A. Weitz. 2004. Elastic behavior of cross-linked and bundled actin networks. *Science.* 304:1301–1305.

88. Lin, Y. C., N. Y. Yao, …, D. A. Weitz. 2010. Origins of elasticity in intermediate filament networks. *Phys. Rev. Lett.* 104:058101.

89. Nikolic, B., E. Mac Nulty, …, G. Wiche. 1996. Basic amino acid residue cluster within nuclear targeting sequence motif is essential for cytoplasmic plectin-vimentin network junctions. *J. Cell Biol.* 134:1455–1467.

90. Andrä, K., B. Nikolic, …, G. Wiche. 1998. Not just scaffolding: plectin regulates actin dynamics in cultured cells. *Genes Dev.* 12:3442–3451.

91. Aufderhorst-Roberts, A., and G. H. Koenderink. 2019. Stiffening and inelastic fluidization in vimentin intermediate filament networks. *Soft Matter.* 15:7127–7136.

92. Hu, J., Y. Li, …, M. Guo. 2019. High stretchability, strength, and toughness of living cells enabled by hyperelastic vimentin intermediate filaments. *Proc. Natl. Acad. Sci. USA.* 116:17175–17180.

93. Wilhelmsen, K., S. H. M. Litjens, …, A. Sonnenberg. 2005. Nesprin-3, a novel outer nuclear membrane protein, associates with the cytoskeletal linker protein plectin. *J. Cell Biol.* 171:799–810.

94. Staszewska, I., I. Fischer, and G. Wiche. 2015. Plectin isoform 1-dependent nuclear docking of desmin networks affects myonuclear architecture and expression of mechanotransducers. *Hum. Mol. Genet.* 24:7373–7389.

Biophysical Journal, Volume *125*

Supplemental information

Plectin affects cell viscoelasticity

at small and large deformations

James P. Conboy, Mathilde G. Lettinga, Nicole van Vliet, Lilli Winter, Gerhard Wiche, Fred C. MacKintosh, and Gijsje H. Koenderink

Plectin affects cell viscoelasticity at small and large deformations

James P. Conboy[1,†], Mathilde G. Lettinga[1,†], Nicole van Vliet[1], Lilli Winter[2], Gerhard Wiche[3], Frederick C. MacKintosh [4], Gijsje H. Koenderink[1,*]

[1]Department of Bionanoscience, Kavli Institute of Nanoscience Delft, Delft, The Netherlands

[3]Department of Cell and Developmental Biology, Center for Anatomy and Cell Biology, Medical University of Vienna, Vienna, Austria

[4]Max Perutz Laboratories, Department of Biochemistry and Cell Biology, University of Vienna, Vienna, Austria

[5]Department of Chemical and Biomolecular Engineering, Rice University, Houston, Texas, United States
Center for Theoretical Biological Physics, Rice University, Houston, Texas, United States
Department of Chemistry and Department of Physics & Astronomy, Rice University, Houston, Texas, United States

[†]These authors contributed equally to this manuscript

[*]Corresponding author: g.h.koenderink@tudelft.nl

1 Supplementary figures

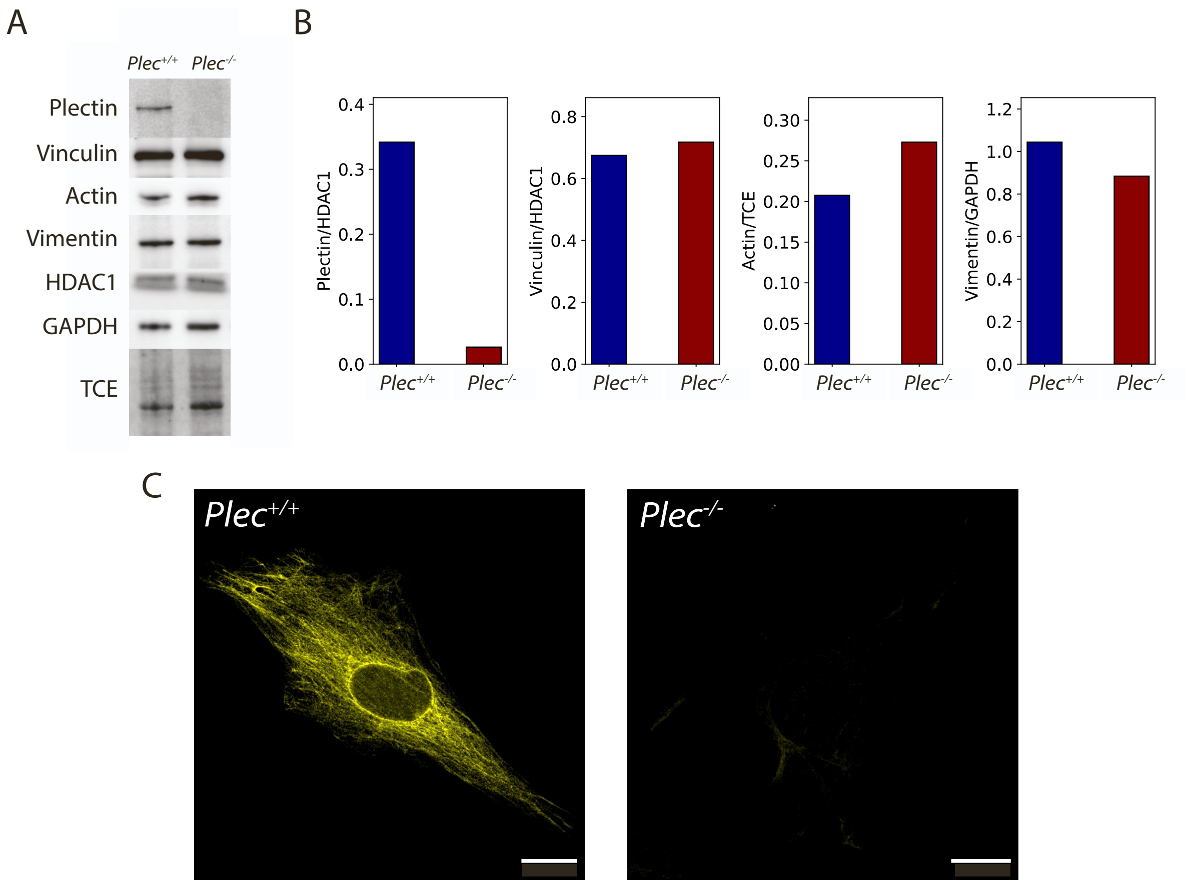

Figure 1: A) Protein expression levels of plectin, vinculin, actin and vimentin in *Plec*$^{+/+}$ and *Plec*$^{-/-}$ mouse embryonic fibroblasts on cropped immunoblots. Depending on the protein of interest, HDAC1, GAPDH or TCE were used as a loading control. B) Quantification of the normalized band intensities as shown in panel A. Data shown for N=1 sample. C) Representative maximum projection immunofluorescent confocal images of *Plec*$^{+/+}$ and *Plec*$^{-/-}$ cells. The images show the absence of plectin (yellow) in *Plec*$^{-/-}$ cells, confirming the results of our immunoblot experiments. Confocal z stacks used to generate the maximum projections shown in the figure were imaged with 7 slices 0.4 µm apart. Scale bar 20 µm.

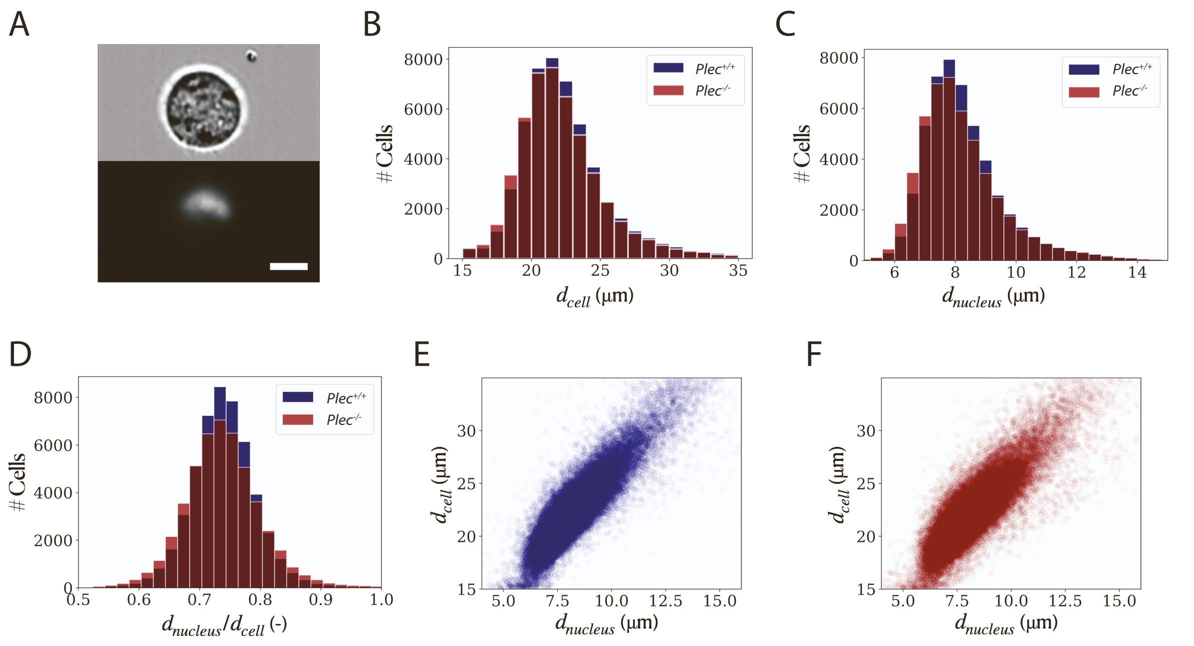

Figure 2: Cell and nucleus sizes measured by imaging flow cytometry for N = 50,220 $Plec^{+/+}$ cells and N = 48,711 $Plec^{-/-}$ cells. (A) Typical unprocessed bright field image of a cell (top) and corresponding epifluorescence image of its nucleus (bottom), which was stained with DRAQ5. Scale bar is 10 μm. (B) The mean cell diameters d_{cell} are 22.4 ± 3.1 μm for $Plec^{+/+}$ cells and 22.2 ± 3.1 μm for $Plec^{-/-}$ cells. (C) Nuclear diameters d_{nucleus} are 8.3 ± 1.4 μm for $Plec^{+/+}$ cells and 8.2 ± 1.4 μm for $Plec^{-/-}$ cells. (D) Nucleus-to-cell diameter ratio, $d_{\text{nucleus}}/d_{\text{cell}}$. On average, the ratio is the same for $Plec^{+/+}$ and $Plec^{-/-}$ cells (0.74 ± 0.06 and 0.74 ± 0.07, respectively). Values stated as mean $\pm$ standard deviation. (E)-(F) Each symbol represents the d_{cell} and d_{nucleus} of a single cell. Both $Plec^{+/+}$ cells (E) and $Plec^{-/-}$ (F) cells display a proportional increase in size with their nucleus size.

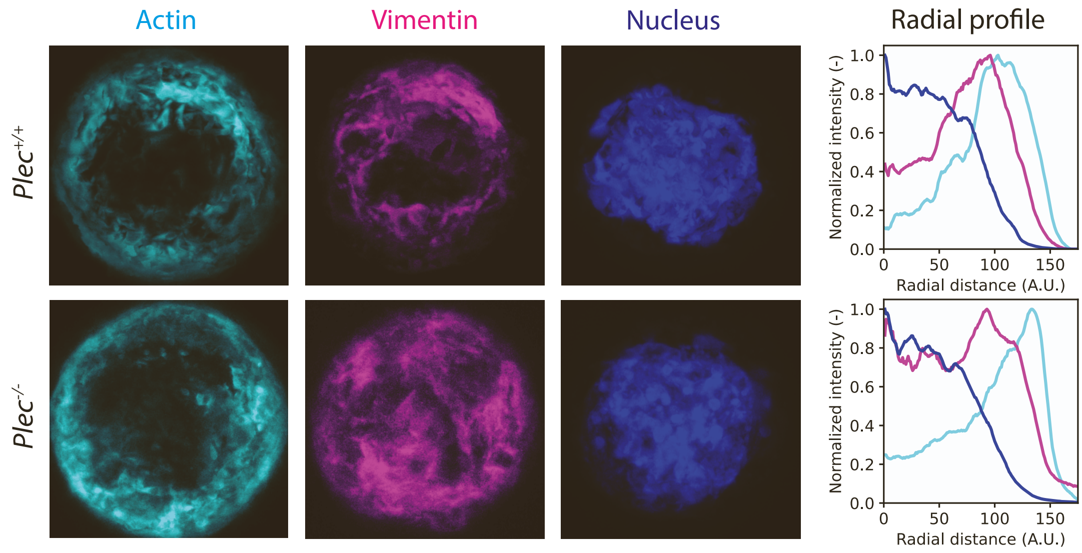

Figure 3: Actin, vimentin and nuclear organization in 'average' $Plec^{+/+}$ cells and $Plec^{-/-}$ cells. As we rescale cells to generate 'average' cells, scale bar is not applicable. The images represent averages of a 2 µm thick confocal section at the cell equator for 10 $Plec^{+/+}$ and 10 $Plec^{-/-}$ cells. For both conditions, filamentous actin is located largely at the cell periphery, while vimentin is located closer to the center of the cell. Average radial profiles of the fluorescent signals (right) were calculated by taking normalized intensities around concentric circles from the center of the image, which show a significant overlapping region between actin and vimentin. The radial distance is set to zero at the cell center; the distance is in arbitrary units (see Methods for details).

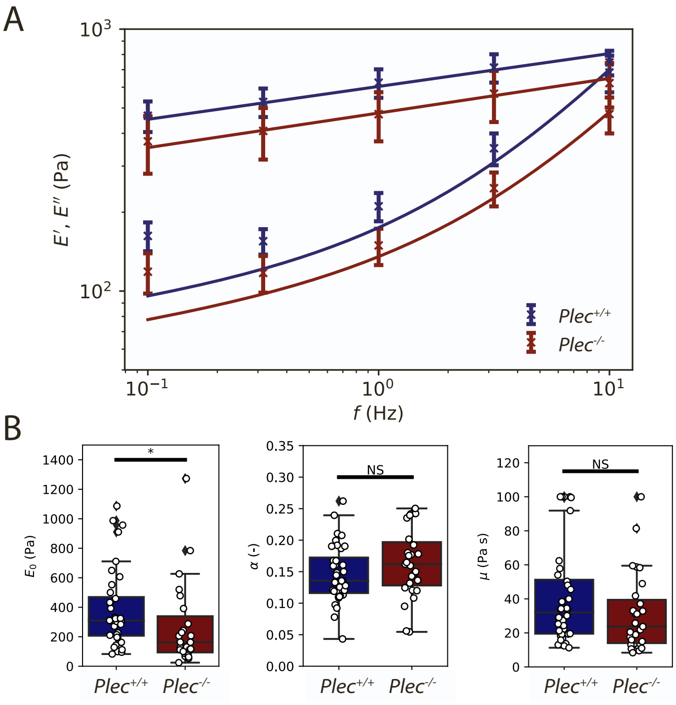

Figure 4: Small amplitude oscillatory strain measurements on single fibroblasts with fits to the structural damping model. (A) Storage (E') and loss (E'') linear compressive moduli (symbols), measured for oscillation frequencies between 0.1Hz and 10Hz for $Plec^{+/+}$ (N=36) and $Plec^{-/-}$ (N=27) cells. Fits of the average data (solid lines) to the structural damping model (equations 1 and 2). The model does a poor job of capturing the mechanical behaviour of these cells at low frequencies. (B) We fit the rheological model to the individual cells to obtain mechanical fingerprints, i.e., scaling factor E_0, power law exponent α and cytoplasmic viscosity μ. Asterisks indicate statistically significant differences (* denotes P $<$ 0.05). N.S. denotes non-significant differences.

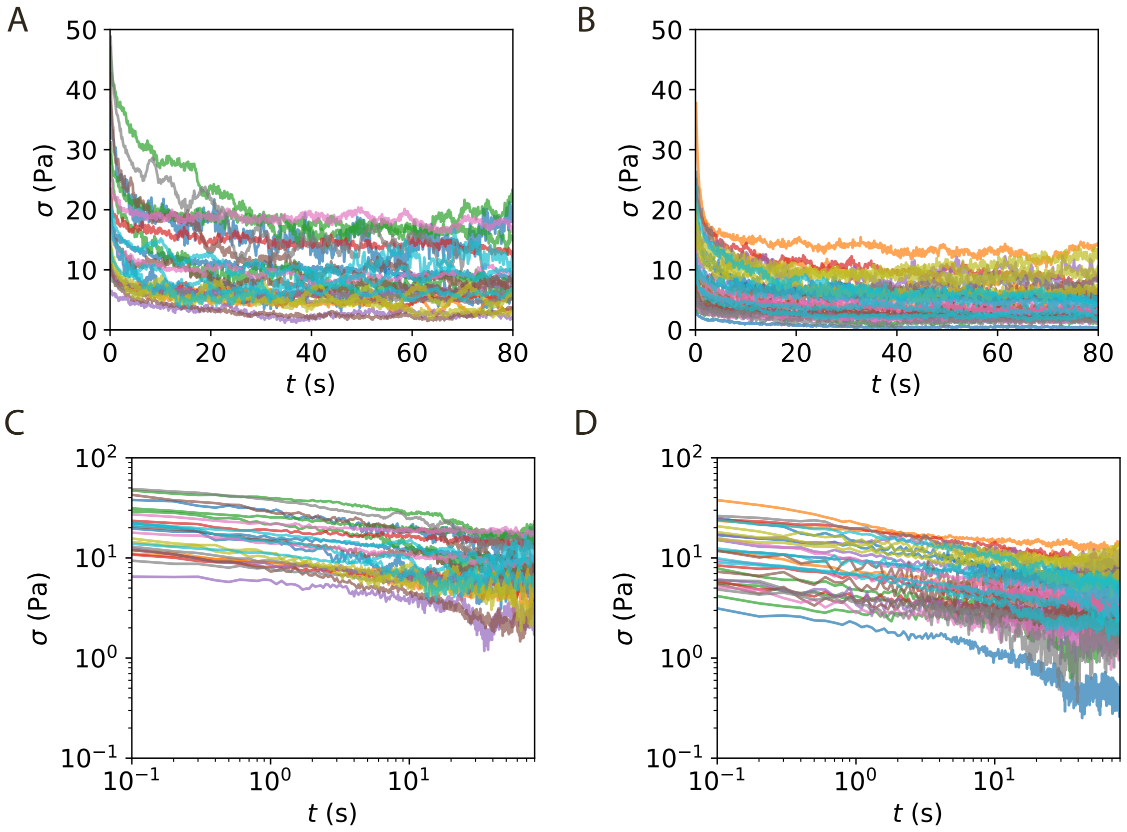

Figure 5: Individual stress relaxation measurements of the mechanical response of $Plec^{+/+}$ and $Plec^{-/-}$ cells to step strain compressions. We rapidly apply a compressive strain and hold it at a fixed value of 0.05. Subsequently, we monitor stress relaxation over 80 s. (A) Stress relaxation for all $Plec^{+/+}$ cells (N=25). (B) Stress relaxation for all $Plec^{-/-}$ cells (N=33). (C) We re-plot (A) on a log-log scale, as shown for averages in the main text. (D) We re-plot (B) on a log-log scale, as shown for averages in the main text.

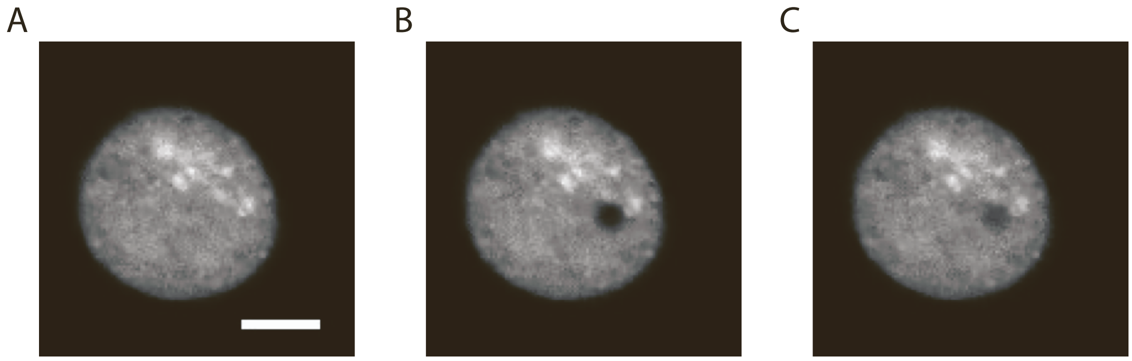

Figure 6: Fluorescence recovery after photobleaching (FRAP) on the cytoplasmic dye CellTracker Orange for a wild type fibroblast confined between two nonadhesive surfaces. The figure shows a representative example of (A) the pre-bleached cell, (B) the cell immediately after bleaching a 1 μm radius circle and (C) partial recovery of the fluorescence signal. Images are shown for a wild type cell. The FRAP experiment was performed in the mid-plane of the cell. Scale bar is 10 μm.

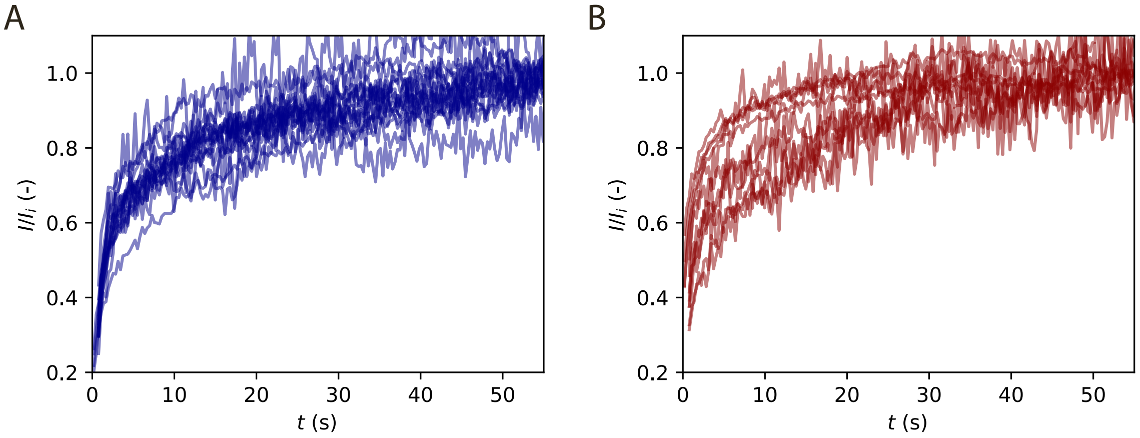

Figure 7: Individual fluorescence recovery curves for the comparison of actin turnover rates for $Plec^{+/+}$ cells (N=54) and $Plec^{-/-}$ cells (N=35) probed by fluorescence recovery after photobleaching (FRAP). (A) Individual normalized intensity curves for $Plec^{+/+}$ cells. (B) Individual curves for $Plec^{-/-}$ cells.

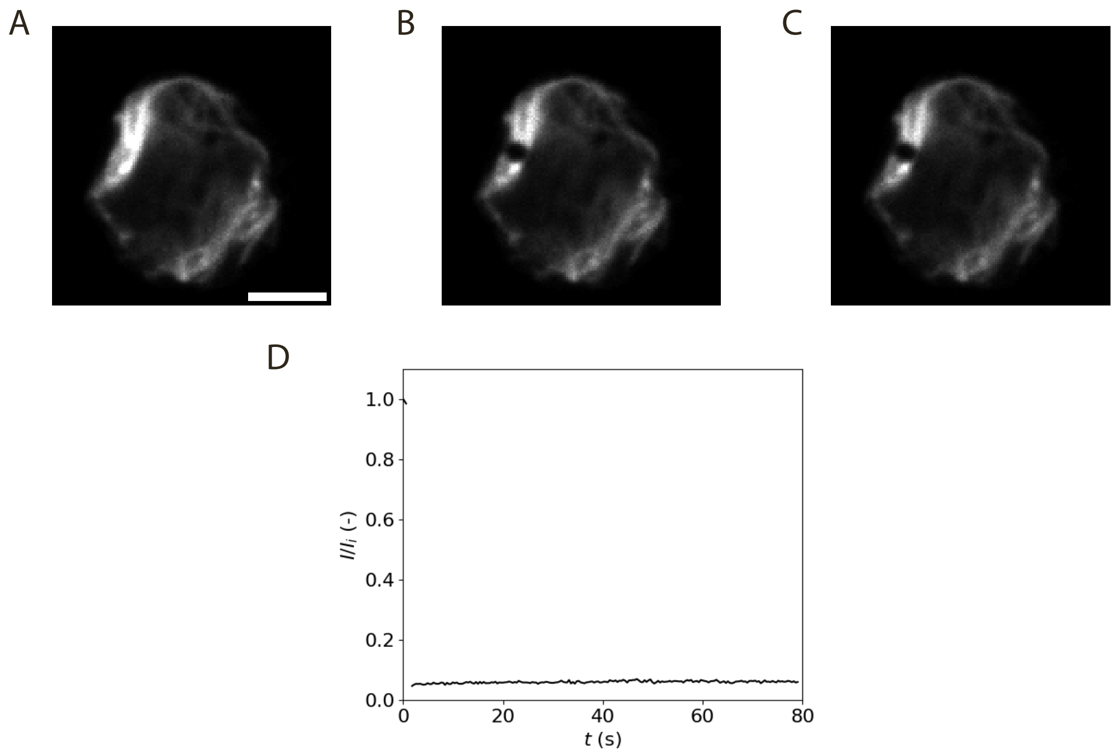

Figure 8: Fluorescence recovery after photobleaching (FRAP) experiments on vimentin-GFP tagged cells. Representative images of a FRAP experiment on a $Plec^{+/+}$ cell (A) before photobleaching, (B) immediately after bleaching a 1 µm radius circle (left hand side), and (C) after 80 s of recovery. The FRAP experiment was performed in the mid-plane of the cell. Scale bar is 10 µm. (D) Representative recovery curve for the vimentin-GFP signal for a $Plec^{+/+}$ cell, showing negligible fluorescence recovery in 80 s (corresponding to the total duration of the stress relaxation experiments).

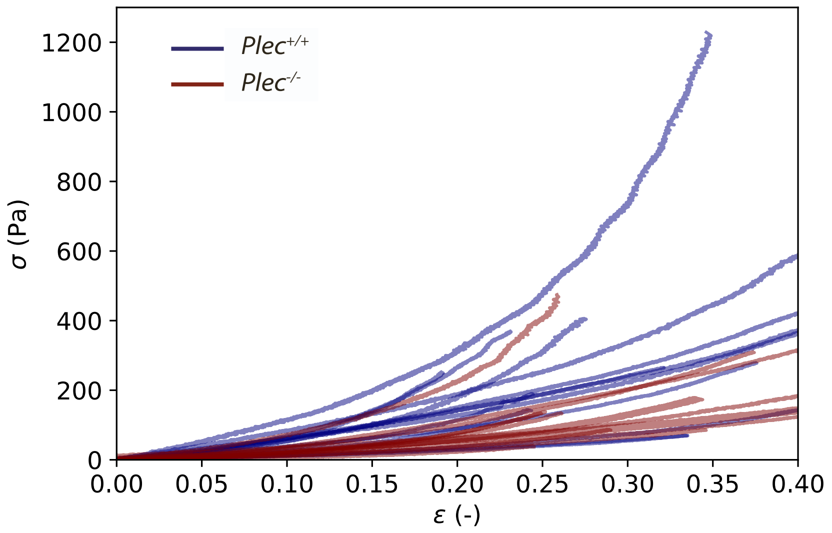

Figure 9: Individual strain ramp measurements of the nonlinear response of *Plec*$^{+/+}$ (blue, N=15) and *Plec*$^{-/-}$ (red, N=13) cells to large compressions, showing stress as a function of strain.

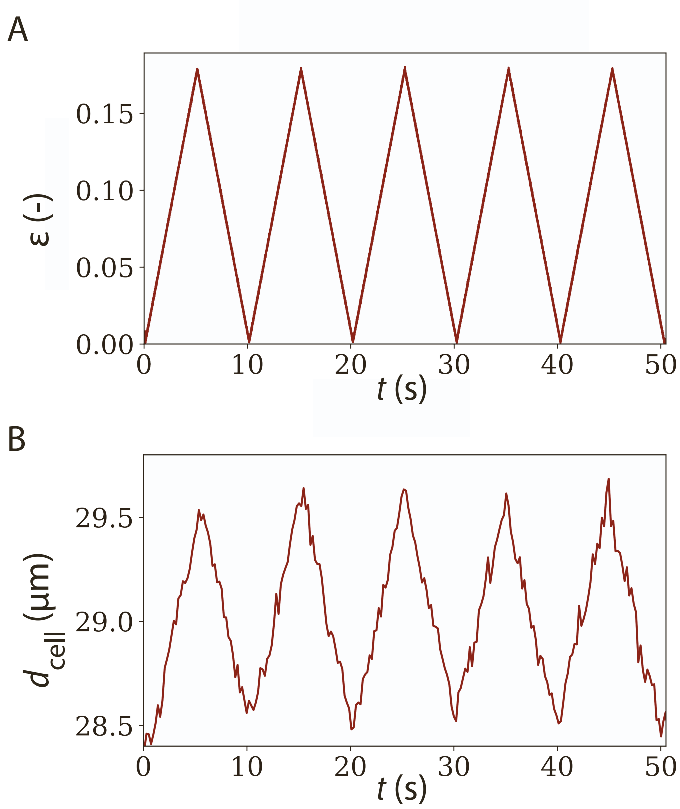

Figure 10: Representative cell diameter of a wild type fibroblast throughout 5 consecutive compressive loading-unloading cycles. (A) Strain profile during five consecutive strain cycles. (B) Measurement of the time-dependent changes in apparent cell diameter determined from the projected cell area observed by wide field epifluorescence imaging of CellTracker Orange. Changes in diameter are reversible.

2 Supplementary tables

**Primary antibody**	**Dilution**	**Manufacturer**	**Catalog number**
Rabbit anti-Plectin IgG	1:1000	Bioke	12254s
Rabbit anti-Vinculin IgG	1:500	Thermofisher	700062
Mouse anti-Actin IgG	1:500	Millipore	MAB1501 Clone C4
Mouse anti-Vimentin IgG1	1:2000	Abcam	AB8978
Rabbit anti-HDAC1 IgG	1:1000	ProteinTech	10197-1-AP
Rabbit anti-GAPDH IgG	1:5000	Cell signal/Bioke	2118s

Table 1: Primary antibodies used for western blot analysis of protein expression levels of $Plec^{+/+}$ and $Plec^{-/-}$ cells.

Secondary antibody	Dilution	Manufacturer	Catalog number
Rabbit anti-mouse IgG (HRP)	1:5000	Abcam	AB6728
Goat anti-rabbit IgG (HRP)	1:5000	Abcam	AB97051

Table 2: Secondary antibodies used for western blot analysis of protein expression levels of $Plec^{+/+}$ and $Plec^{-/-}$ cells.

3 Supplementary methods

Immunofluorescence imaging of plectin

Cells were plated on glass coverslips. 24 hours after plating, cells were fixed with ice-cold methanol for 90 seconds at -20°C and immunostained using guinea pig antiserum to plectin (GP-21, Progen Biotechnic) and goat antiserum to vimentin (P. Traub, University of Bonn, Bonn, Germany) in combination with biotinylated rabbit-anti guinea pig IgG together with streptavidin conjugated Alexa Plus 488 and donkey anti-goat IgG Alexa Fluor Plus 555 (all from Invitrogen). Nuclei were visualized with DAPI (Sigma-Aldrich). Microscopy was performed using an Olympuis FLUOVIEW FV3000 confocal microscope equipped with a PlanApo N 60x 1.4 NA objective lens (Olympus).

Fitting the structural damping law to oscillatory strain measurements

Before fitting the fractional viscoelastic model described in the main text, we first tried to capture the rheology of our cells using a simpler phenomenological model. This is a three parameter model referred to as the structural damping model [1–3], described in the main text as Equation 1. We take this equation and decompose it into its respective real and imaginary parts to yield the storage (E') and loss (E'') moduli which we fit to our data:

$$E'(f) = E_0 c(\alpha) \cos\left(\frac{\pi\alpha}{2}\right)\left(\frac{f}{f_0}\right)^{\alpha} \tag{1}$$

$$E''(f) = E_0 c(\alpha) \sin\left(\frac{\pi\alpha}{2}\right)\left(\frac{f}{f_0}\right)^{\alpha} + \mu f. \tag{2}$$

The three fitting parameters are the scaling factor E_0, power law exponent α, and cytoplasmic viscosity μ. Using the curve fit function from Python's SciPy library [4], we simultaneously fitted Equations 1 and 2 to the measured storage and loss moduli.

Determination of cell and nucleus size by imaging flow cytometry

An ImageStreamX Mark II Imaging Flow Cytometer with a 40x objective was used to measure the sizes of the cells and their nuclei. One day before cytometry experiments, cells were detached with Trypsin-EDTA (0.25%) (25200056 Thermo Fisher Scientific) and counted using a Countess cell counter (Thermo Fisher Scientific). Next, 100,000 cells were transferred to plastic cell culture 6-well plates (83.3920.005 Sarstedt) containing culture medium. On the day of experiments, cell nuclei were fluorescently stained with DRAQ5 (P5739873 ThermoFisher) by adding DRAQ5 from a 5 mM stock solution in DMSO to the culture medium (1:500), and leaving the cells to incubate for 30 min. The cells were imaged in bright field, while the DRAQ5-stained nuclei were imaged in fluorescence with a 642 nm laser. The flow rate was set to Low in the ImageStreamX software, with High sensitivity, amounting to a flow speed of 55 mm/s. Single cells were separated from debris and cell clusters by selecting only objects with a diameter between 10 µm and 35 µm, a nuclear area of 50-5000 μm^2, and a cellular aspect ratio of 0.7-1. A typical image taken with the ImageStream flow cytometer is shown in Figure S2A. From the projected areas of the cells and nuclei, the corresponding average diameters were calculated, assuming spherical shapes in an automated Python script.

Monitoring the cell diameter during repeated ramp experiments

For experiments measuring the cell diameter during repeated compression, we used 35 mm glass bottom dishes (81218-200 Ibidi), in combination with a 40x NA 1.10 water immersion objective. To determine the cell diameter, we recorded each cell at a frame rate of 5fps. To determine the cell area, we thresholded each frame using Otsu's method in the Python library scikit image [5] and multiplied the number of pixels by the pixel size in the thresholded image. To determine the cell diameter, we used the geometric relation between a circle diameter and area: $A = \pi(d_{\text{cell}}/2)^2$.

Supplementary results

Flow cytometry

Complementing the images in the main text, we performed imaging flow cytometry measurements to determine cellular and nuclear sizes. The corresponding histograms are shown in Figure S2B-D. On average, *Plec*$^{-/-}$ cells have a diameter of 22.2 ± 3.1 µm, and are thus slightly smaller than *Plec*$^{+/+}$ cells with diameter 22.4 ± 3.1 µm. This diameter is calculated from the projected area a as $d_{\mathrm{cell}} = 2\sqrt{a/\pi}$. In Figure S2B, we see that the diameters follow similar distributions, which appear to be log-normal distributions. Diameters range from 15 µm to 35 µm. The distributions for the nuclear sizes are shown in Figure S2C, where the diameter was again determined from the projected area. The resulting mean diameters d_{nucleus} are 8.3 ± 1.4 µm for *Plec*$^{+/+}$ cells and 8.2 ± 1.4 µm for *Plec*$^{-/-}$ cells. Dividing d_{nucleus} by d_{cell} for each cell yields the distribution shown in Figure 2D, with an average ratio of 0.74 ± 0.06 for *Plec*$^{+/+}$ cells and 0.74 ± 0.07 for *Plec*$^{-/-}$ cells. The ratios are thus comparable and both display a Gaussian distribution, with a slightly wider distribution for *Plec*$^{-/-}$ cells. This hints at a scaling between nucleus and cell size, which we confirmed by making scatter plots (Figure 2E-F).

Estimation of the poroelastic diffusion constant assuming fluid transport through the nucleus

In the main text, we make an estimate of the poroelastic diffusion constant, where we assume that the relevant length scale of fluid transport in the cell (L) is that of the cell membrane to the nucleus. If we instead use the cell size as the relevant length scale (i.e., assuming fluid transport occurs through cytoplasm and nucleus), we estimate significantly larger values for D_{p} of (1700 ± 100) µm^2/s for *Plec*$^{+/+}$ cells and (3200 ± 200) µm^2/s for *Plec*$^{-/-}$ cells. Importantly, we still observe a larger poroelastic diffusion constant in *Plec*$^{-/-}$ cells.

References

[1] Ben Fabry, Geoffrey N. Maksym, James P. Butler, Michael Glogauer, Daniel Navajas, and Jeffrey J. Fredberg. Scaling the microrheology of living cells. *Physical Review Letters*, 87(14):148102, 2001.

[2] Sebastian Hurst, Bart E. Vos, Matthias Brandt, and Timo Betz. Intracellular softening and increased viscoelastic fluidity during division. *Nature Physics*, 17(11):1270–1276, 2021.

[3] A. Bonfanti, J. L. Kaplan, G. Charras, and A. Kabla. Fractional viscoelastic models for power-law materials. *Soft Matter*, 16(26):6002–6020, 2020.

[4] Pauli Virtanen, Ralf Gommers, Travis E. Oliphant, Matt Haberland, Tyler Reddy, David Cournapeau, Evgeni Burovski, Pearu Peterson, Warren Weckesser, Jonathan Bright, Stéfan J. van der Walt, Matthew Brett, Joshua Wilson, K. Jarrod Millman, Nikolay Mayorov, Andrew R.J. Nelson, Eric Jones, Robert Kern, Eric Larson, C. J. Carey, İlhan Polat, Yu Feng, Eric W. Moore, Jake VanderPlas, Denis Laxalde, Josef Perktold, Robert Cimrman, Ian Henriksen, E. A. Quintero, Charles R. Harris, Anne M. Archibald, Antônio H. Ribeiro, Fabian Pedregosa, Paul van Mulbregt, Aditya Vijaykumar, Alessandro Pietro Bardelli, Alex Rothberg, Andreas Hilboll, Andreas Kloeckner, Anthony Scopatz, Antony Lee, Ariel Rokem, C. Nathan Woods, Chad Fulton, Charles Masson, Christian Häggström, Clark Fitzgerald, David A. Nicholson, David R. Hagen, Dmitrii V. Pasechnik, Emanuele Olivetti, Eric Martin, Eric Wieser, Fabrice Silva, Felix Lenders, Florian Wilhelm, G. Young, Gavin A. Price, Gert Ludwig Ingold, Gregory E. Allen, Gregory R. Lee, Hervé Audren, Irvin Probst, Jörg P. Dietrich, Jacob Silterra, James T. Webber, Janko Slavič, Joel Nothman, Johannes Buchner, Johannes Kulick, Johannes L. Schönberger, José Vinícius de Miranda Cardoso, Joscha Reimer, Joseph Harrington, Juan Luis Cano Rodríguez, Juan Nunez-Iglesias, Justin Kuczynski, Kevin Tritz, Martin Thoma, Matthew Newville, Matthias Kümmerer, Maximilian Bolingbroke, Michael Tartre, Mikhail Pak, Nathaniel J. Smith, Nikolai Nowaczyk, Nikolay Shebanov, Oleksandr Pavlyk, Per A. Brodtkorb, Perry Lee, Robert T. McGibbon, Roman Feldbauer, Sam Lewis, Sam Tygier, Scott Sievert, Sebastiano Vigna, Stefan Peterson, Surhud More, Tadeusz Pudlik, Takuya Oshima, Thomas J. Pingel, Thomas P. Robitaille, Thomas Spura, Thouis R. Jones, Tim Cera, Tim Leslie, Tiziano Zito, Tom Krauss, Utkarsh Upadhyay, Yaroslav O. Halchenko, and Yoshiki Vázquez-Baeza. SciPy 1.0: fundamental algorithms for scientific computing in Python. *Nature Methods*, 17(3):261–272, 2020.

[5] Stéfan Van Der Walt, Johannes L. Schönberger, Juan Nunez-Iglesias, François Boulogne, Joshua D. Warner, Neil Yager, Emmanuelle Gouillart, and Tony Yu. Scikit-image: Image processing in python. *PeerJ*, 2014(1):1–18, 2014.
